# Strategies to Apply Water-Deficit Stress: Similarities and Disparities at the Whole Plant Metabolism Level in *Medicago truncatula*

**DOI:** 10.3390/ijms22062813

**Published:** 2021-03-10

**Authors:** Verónica Castañeda, Esther M. González

**Affiliations:** Department of Sciences, Institute for Multidisciplinary Research in Applied Biology, Public University of Navarra, E-31006 Pamplona, Spain; veronica.castaneda@unavarra.es

**Keywords:** Water stress, phloem sap, PEG, salt stress, proline, carbon metabolism, ionome, glutathione

## Abstract

Water-deficit stresses such as drought and salinity are the most important factors limiting crop productivity. Hence, understanding the plant responses to these stresses is key for the improvement of their tolerance and yield. In this study *M. truncatula* plants were subjected to 250 mM NaCl as well as reduced irrigation (No-W) and 250 g/L polyethylene glycol (PEG)-6000 to induce salinity and drought stress, respectively, provoking a drop to −1.7 MPa in leaf water potential. The whole plant physiology and metabolism was explored by characterizing the stress responses at root, phloem sap and leaf organ level. PEG treatment led to some typical responses of plants to drought stress, but in addition to PEG uptake, an important impairment of nutrient uptake and a different regulation of carbon metabolism could be observed compared to No-W plants. No-W plants showed an important redistribution of antioxidants and assimilates to the root tissue, with a distinctive increase in root proline degradation and alkaline invertase activity. On the contrary, salinity provoked an increase in leaf starch and isocitrate dehydrogenase activity, suggesting key roles in the plant response to this stress. Overall, results suggest higher protection of salt-stressed shoots and non-irrigated roots through different mechanisms, including the regulation of proline and carbon metabolism, while discarding PEG as safe mimicker of drought. This raises the need to understand the effect at the whole plant level of the different strategies employed to apply water-deficit stress.

## 1. Introduction

Plant growth, development and productivity are adversely affected by various abiotic stress factors, drought and soil salinity being the major problems, reducing crop productivity by more than 50% [1]. It is then of outmost importance to breed crops capable of tolerating or resisting these stresses without a significant impact on their yield. However, the knowledge about drought and salinity stress resistance is still limited, partly due to the complexity of the trait, involving important metabolic and physiological changes at the whole plant level. Water deficit can be defined as any water content of a tissue or cell below the highest water content exhibited in the most hydrated state. Hence, although the terms “drought stress” and “water-deficit stress” are usually employed indistinctively, water does not only become limiting for plant communities as a result of inadequate rainfall but also due to other environmental conditions like excessive salinity in the soil solution. Both drought and salinity cause osmotic stress by lowering the water potential of plant cells, which can lead to cell turgor loss, membrane disorganization, protein denaturation, inhibition of photosynthesis and oxidative damage, and thus both stresses share many plant responses [2]. However, in addition to the cellular water deficit provoked by the presence of salts, salinity stress also has an ionic component that further alters plant ion homeostasis through Na^+^ and Cl^−^ toxicity.

Both water-deficit stresses affect plant growth and development through changes in the plant biochemistry, physiology and morphology which have been investigated independently in many plant species, including *Medicago truncatula* [3,4]. Studies comparing different osmotic stresses are much less common, with a few done in some crop plants such as *M. falcate* [5] and rice [6]. In *M. truncatula*, Staudinger et al. observed significant differences in the degree and strategy of early drought, as compared to salt stress response, concluding that N-nutrition seems of crucial importance for plant stress acclimation [7]. However, more studies are needed in order to better understand the plant response to different water-deficit stresses. It is not surprising that no irrigation and salinity stress lead to a severe alteration of the carbon and nitrogen metabolism of the plant, which are the main providers of energy and nutrients of the plant [8] The coordination of carbon and nitrogen assimilation ensures the availability of amino acids and carbon skeletons, building blocks of biomass production and important compounds associated with increased water-stress tolerance, at the required amounts and moment [9]. However, despite their high relevance on plant growth and stress tolerance, their response to water-deficit stress is still not fully understood.

The transport of carbon and nitrogen assimilates occurs in the phloem, a part of the vascular system that connects all plant parts and directs the movement of these metabolites from their sites of synthesis (“sources”, such as mature leaves) to the sites of utilization or “sinks” (heterotrophic organs such as roots or growing leaves) [10]. The most accepted mechanism for phloem transport drive is explained by an osmotic pressure differential between the source and sink tissues. This implies that changes in the sink and/or source tissues such as those triggered by abiotic stresses can alter phloem transport and, hence, vital aspects such as growth, stress tolerance and reproduction [11,12]. Despite the crucial role of the phloem as essential metabolite transporter and its impact on plant–environment interactions, little research has been done on how its potential function alteration influences plant response to stress [13]. However, compelling studies suggest that the response of the phloem sap to stress conditions such as drought might predict plant survival and recovery capacity and can, in any case, help us better understand the response to stress at a whole-plant level [14]. To our knowledge and up to this date, only one study has been conducted in *M. truncatula* considering the phloem sap and its response to water-deficit stress [15]. In this proteomics study, Castañeda et al. described the existence of a core stress responsive proteome in *M. truncatula* across different tissues including the phloem sap, suggesting a major role of the phloem in stress protection and antioxidant activity, especially by linking below and above ground communication in order to fine-tune stress response [15]. Other studies considering the phloem sap and different water-deficit stresses have been made in tomato [16,17], *Plantago major* [18], white clover [19], *Ricinus communis* [20], maize [21] and alfalfa [22].

Many authors have studied the effect of water-deficit stress in leaves [23] or roots [24,25], but not many have simultaneously addressed the response in both organs [26,27], and even less at the whole-plant level including the phloem sap [15,19], even though the study of a plant as a whole can give us a unique and valuable information and understanding of its response to stress. We hypothesize that the impact of different osmotic treatments frequently applied to simulate water-deficit stress is markedly different at the whole-plant level. Here we assess the effect of different treatments (250 mM NaCl, 250 g/L polyethylene glycol (PEG)-6000 and reduced irrigation (No-W)) in roots, leaves and phloem sap of *M. truncatula* plants to better understand the response to different kinds of water-deficit mimickers. Overall, results suggest a higher protection of shoots in salt stressed plants while roots are the major responding organ under no-irrigation conditions. On the other hand, PEG caused an important impairment of nutrient uptake, which dismisses a confident use to mimic water-deficit stress.

## 2. Results

### 2.1. Physiological Measurements

NaCl, no-watering (No-W) and PEG-6000 stress were applied to provoke a drop in the leaf water potential similar to a moderate water-deficit level. Selected treatments were based on results of previous studies, in which the addition of 250 mM NaCl and 250 g/L PEG-6000 led to an average drop to Ψ_leaf_ −1.7 MPa after 7 days. Visually, salt-treated and No-W plants showed a vigorous and turgid appearance in the shoots, but some yellowing could be observed in the oldest leaves of salt-treated plants. On the other hand, PEG treated plants showed a less leafy appearance and a whitish discoloration in the leaves (Figure S1). Regarding plant biomass, the imposition of water-deficit stress did not significantly impact total, shoot or root dry biomasses except for a higher root/shoot ratio in No-W plants compared to the other treatments (Table 1). 

Control plants exhibited a Ψ_leaf_ value of −0.50 ± 0.00 MPa, while the NaCl, PEG and No-W stresses led to drops in the Ψ_leaf_ to an average value of −1.70 ± 0.02 MPa. The stomatal conductance was severely reduced under NaCl stress, while this parameter reached values close to zero in No-W and PEG treated plants. Accordingly, transpiration rates were also markedly decreased under all stress treatments, with no significant differences among them. On the other hand, the chlorophyll content, measured in the second expanded leaf, decreased only slightly in the PEG treatment. It should be noted that no measurements were made in the oldest leaves. Leaf water content (WC) did not significantly decrease in neither salt nor No-W treatments, while dropping around 13.6% in the PEG treatment. Conversely, the No-W stress was the only treatment significantly reducing from 82.4% to around 65% the root water content (Table 1). On the other hand, the average PEG concentration measured in leaves and roots of PEG treated plants was 8.24 ± 1.33 and 152.88 ± 16.49 mg g dry weight (DW^−1^), respectively.

### 2.2. Carbon Metabolites

The concentration of starch and soluble sugars was differently affected by the stress treatments in the leaf, root and phloem sap of *Medicago truncatula* (Table 2). Under NaCl stress starch and sucrose accumulated markedly in leaves, being mostly unaffected or slightly modified in roots and phloem. However, in PEG-treated plants, starch was fully depleted in leaves and significantly reduced in roots, while sucrose accumulated markedly in leaves and roots without any change at phloem sap level. A significant decrease in starch was observed in the No-W treated plants concomitant with a moderate accumulation of sucrose. Both salt and PEG treatments led to a depletion of glucose and fructose at root level, being unaffected at the leaf level. Conversely, No-W treatment showed a marked hexose increase in leaves and roots.

Regarding organic acids, malate and citrate were more abundant than succinate and α-ketoglutarate (α-KG), both in leaves and roots. NaCl stress significantly affected malate, citrate and succinate content in leaves, while provoking an overall decrease in the malate content on the root tissue. In salt-stressed plants, the α-KG level also decreased in the root tissue, whilst it accumulated significantly in leaves. No-W stress had a minor impact on organic acids: the more abundant organic acids, malate and citrate, were not significantly affected in either leaves or roots, while α-KG increased slightly in the latter. PEG treatment led to a decline in citrate and succinate levels in the shoots, while a significant decrease in citrate was observed in roots together with an up to six-fold accumulation of succinate levels (Table 2). 

### 2.3. Nitrogen Status

The soluble protein content (Table 3) was not significantly affected by any treatment in leaves while it showed a decreasing trend in roots. A significant increase in total free amino acid concentration was found in leaves and roots of the PEG and No-W treatments whilst this parameter was not significantly affected by salinity (Table 3). The amino acid content of the phloem sap exhibited a marked increase in NaCl and PEG treatments. Although the No-W treatment significantly altered the total amino acid concentration of leaves and, above all, roots, it did not show any changes at phloem level. Proline (Pro) accumulated in all the treatments and organs, including the phloem sap. In NaCl-treated plants, Pro increased around 14-fold in leaves and roots with a remarkable accumulation at the phloem level (24-fold). Pro accumulation was much less marked in the leaves of No-W treated plants, while the highest increase (29-fold) was observed at the root level. PEG provoked a similar Pro accumulation in leaves and roots (30-fold) with a more moderate increase at the phloem level. Complete data of individual amino acids are shown in Table S1. In general, the concentration of leaf amino acids, except for Pro, was not markedly affected by any stress, the leaves of No-W treated plants being the most affected tissue. No-W also showed the most altered root amino acid content, even though PEG showed an important alteration too, including the accumulation of the branched-chain amino acids (BCAA) Leu, Ile and Val. Conversely, the higher phloem sap alteration occurred under salt stress, where Pro, GABA, Ser, Gln and Ala showed significant increments and Val declined.

### 2.4. Inorganic Ions

**Figure 1:**
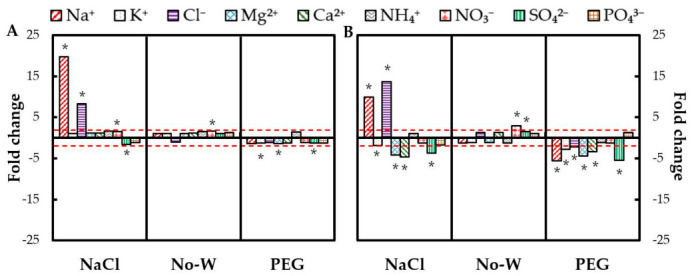
Representation of the inorganic ion content response to water-deficit stress. Bars represent the means (*n* = 5) of the treatment/control ratios fold change in leaves (**A**) and roots (**B**). Asterisks represent statistical differences (Student’s *t*-test, *p* < 0.05) between control and treatments. No-W, no-watering.

Figure 1A,B show the treatment:control fold change for each ion in leaves and roots in response to the different treatments. NaCl stress provoked a marked accumulation of both ions in leaves and roots, Na^+^ being preferentially accumulated in leaves and Cl^−^ in roots. However, irrigation with NaCl also lead to a decrease in K^+^, Mg^2+^, Ca^2+^ and SO_4_^2−^ levels in the roots. PEG provoked an ion alteration mostly at the root level, leading to a decline in the Na^+^, K^+^, Mg^2+^, Ca^2+^ and SO_4_^2−^ concentrations. Lastly, No-W did not significantly modify the inorganic ions levels in any tissue, and only NO_3_^−^ and SO_4_^2−^ exhibited a significant accumulation in roots. The absolute ion content under all stresses and both leaves and roots can be found in Table S2. In addition to the already stated changes in the ionic content, a remarkable increase in total ion content could be observed in both tissues in response to salinity stress, while the total ion content was decreased under PEG-6000.

### 2.5. Antioxidant Content

Ascorbate, glutathione and homoglutathione total contents in the different tissues and treatments are shown in Table 4, while complete data including their respective reduced and oxidized forms can be found in Table S3. The antioxidant metabolites’ concentration in the phloem sap was below the detection limit of our analytical system (5 ppm). Salt stress did not significantly modify the total glutathione or homoglutathione pools but provoked a significant decrease in the total ascorbate pool. Similarly, in leaves of No-W treated plants, total glutathione and homoglutathione levels were maintained stable but the ascorbate pool decreased significantly, although less than under salinity. Conversely, in roots, total glutathione and homoglutathione significantly increased concomitantly with the decrease in the ascorbate pool. PEG provoked an overall decrease in the ascorbate pool in leaves and roots, while the glutathione and homoglutathione pools remained unaffected. Since the total ascorbate level was near the detection level of our analytical method in the root tissue, it was not possible to discern between both antioxidant forms in this tissue in those treatments provoking a decrease in the ascorbate pool such as No-W or PEG, and this metabolite could then only be measured as the whole pool. Regarding the ratio reduced form/total pool for each metabolite, the ascorbate and glutathione ratios in leaves showed a decreasing trend, while the reduced homoglutathione/total homoglutathione ratio showed an increasing trend in response to all treatments. Conversely, in roots, the glutathione ratio showed a decreasing trend, while the homoglutathione ratio remained unaffected under all conditions.

### 2.6. Carbon and Nitrogen Enzymatic Activities

Several enzymatic activities related to the carbon [alkaline invertase (alkINV), glucose-6-phosphate dehydrogenase (G6PDH), isocitrate dehydrogenase (IDH), sucrose synthase (SuSy)], nitrogen metabolism [aspartate aminotransferase (AAT), glutamate dehydrogenase (GDH), glutamate synthase (GOGAT) and glutamine synthase (GS)] and proline [ornithine aminotransferase (OAT), Δ^1^-1-pyrroline-5-carboxylate synthase (P5CS) and proline dehydrogenase (ProDH)] metabolism were measured in leaves and roots of *M. truncatula* (Table 5). 

Overall, the measured carbon and nitrogen-related enzyme activities were not significantly affected in leaves, except for an increase in the IDH activity under salinity stress. On the contrary, the root tissue showed a generalized response at enzymatic activity level: AAT and SuSy activities showed a general down-regulation in all treatments. Conversely, alkINV showed a significant enhancement in the No-W treatment while it decreased in the PEG-treated roots. Root GOGAT, GDH and IDH activities did not significantly change in response to any treatment.

Proline synthesis-related P5CS and OAT enzymes increased significantly in leaves and roots of salt-stressed plants, while the proline-degrading ProDH was slightly inhibited in the leaves (Table 5). However, in leaves of No-W treated plants proline metabolism was not affected while the three enzymatic activities increased significantly in roots. This Pro metabolism activation occurred also in roots of PEG-treated plants, while OAT was negatively affected in leaves. 

### 2.7. Principal Component Analysis

**Figure 2:**
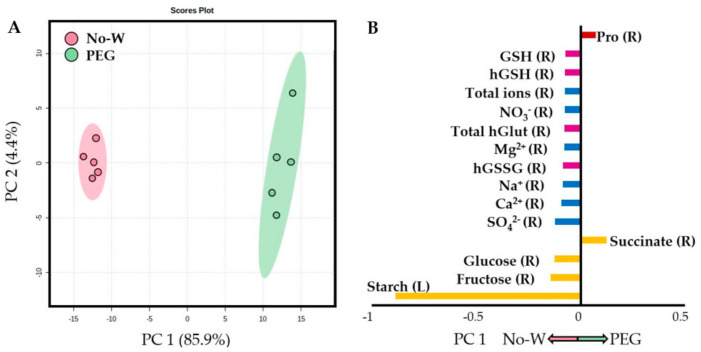
Principal Component Analysis (PCA) conducted with all measured parameters in No-W and PEG-6000 treated plants. (**A**) PCA scores plot showing the non-irrigated (No-W) and PEG-6000 cases. (**B**) Loading values of all variables in PC1 separating both treatments. GSH, reduced glutathione; hGlut, homoglutathione; hGSH, reduced homoglutathione; hGSSG, oxidized homoglutathione; L, leaves; R, roots. Bar key: yellow, carbohydrates; blue, inorganic ions; pink, antioxidants; red, amino acids.

**Figure 3:**
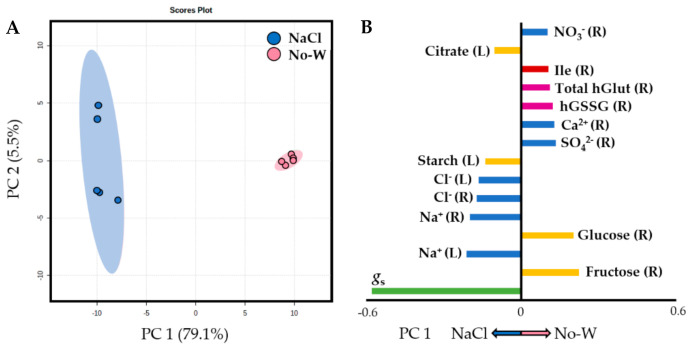
Principal Component Analysis conducted with all measured parameters in NaCl and No-W treated plants. (**A**) PCA plot showing the NaCl and non-irrigated (No-W) cases. (**B**) Loading values of all variables in PC1 separating both treatments. *g*_s_, stomatal conductance; hGlut, homoglutathione; hGSSG, oxidized homoglutathione; L, leaves; R, roots. Bar key: green, physiological measurements; yellow, carbohydrates; blue, inorganic ions; pink, antioxidants; red, amino acids.

A Principal Component Analysis (PCA) was performed with all measured parameters in order to further confirm the differences between the treatments. All treatments were compared in Figure S2, in which a clear differentiation between samples, above all between control and drought treatments, could be observed. Indeed, No-W- and PEG-treated plants showed different scores along the first PC axis (Figure 2A), with the variables with the most weight responsible for the separation of both treatments being leaf starch, root fructose and glucose contents, root succinate, root Na^+^, Ca^2+^, Mg^2+^, NO_3_^−^, SO_4_^2−^ and total ion contents, as well as root oxidized, reduced and total homoglutathione, root reduced glutathione and leaf proline contents (Figure 2B). A very obvious sample separation occurred when NaCl and No-W treated plants were analyzed (Figure 3A), with the most responsible variables being leaf stomatal conductance, root fructose and glucose contents, leaf and root Na^+^ and Cl^−^ contents, leaf starch and citrate, root SO_4_^2−^, NO_3_^−^ and Ca^2+^ contents, root Ile and root oxidized and total homoglutathione contents (Figure 3B). The statistical significance of the cited parameters was further acknowledged by Student’s *t*-test (*p* < 0.05), while the complete list of loading values for each PCA is shown in Table S4. All measurements in performed in this study and used for the PCA analyses can be found in Table S5.

## 3. Discussion

### 3.1. PEG: A False Mimetic of Drought

PEG is a polymer widely employed to mimic drought stress. One of the advantages of using this osmotic agent versus water-deficit imposition through withholding irrigation is the possibility to adjust precisely the stress level in the hydroponic solution, yielding a more uniform and controlled stress [28]. However, even though PEG-6000 molecules are usually described as too large to be taken up by intact roots, several studies have observed PEG uptake by both leaves and roots [29,30,31,32]. This, together with the hypoxic nature of the highly-viscous PEG solution, can lead to additional stresses in plants subjected to PEG irrigation [33].

For this study, PEG treated plants exhibiting a Ψ_leaf_ ≈ −1.7 MPa were selected in order to perform a comparative analysis with No-W treated plants at an equal level of water-deficit stress. In this context, we found some common physiological and metabolic responses such as the reduced transpiration and stomatal conductance (Table 1) and root soluble protein content, as well as the general amino acid accumulation, particularly proline (Table 3). These responses are in agreement with previous studies with plants subjected to drought stress as part of their drought stress response [34]. However, PEG treated plants’ water content behaved in a completely opposite way compared to No-W treated plants, with a lower leaf and higher root water content under PEG (Table 1). This is in agreement with Fan and Blake, who observed a higher decline in leaf RWC of 5-month-old seedlings of jack pine, black spruce and flooded gum treated with PEG compared to those subjected to no irrigation [35]. This could be a sign of PEG uptake and/or hypoxia, which have been reported to disturb leaf water relations by blocking the water conducting channels and possibly exert a toxic response [31,33,35,36].

One of the most divergent aspects differentiating no irrigation and PEG treatment was the carbohydrate content (Table 2, Figure 2). Indeed, even though starch depletion has been a long time described phenomenon under drought stress that serves as energy and carbon supply when photosynthesis may be limited [37], its decline was more dramatic under PEG conditions. In addition, sucrose accumulation was especially remarkable in leaves of PEG-treated plants, while hexoses concentration only increased in No-W leaves (Table 2, Figure 2). This increase in leaf soluble sugars has been described as a common behavior in drought-stressed plants due to their role in osmoregulation, amongst others [34]. Darko et al. also reported a different pattern of soluble sugars accumulation in leaves and roots of wheat seedlings depending on the studied stress even under iso-osmotic conditions [38], which could have a role in the signaling process of the regulation of plant metabolism and developmental processes [38,39,40].

Proline accumulation is another drought stress response that is observed early in the onset of osmotic stress [34]. In our study, both PEG and No-W treated plants showed a marked increase in this compatible solute, with a much higher accumulation in PEG treated leaves and phloem sap (Table 3, Figure 2). Cui et al. observed a similar accumulation of proline under soil drought and PEG stress consistent with the increases in the content of key enzymes involved in proline metabolism [40]. However, like in our study, a differential activation of proline metabolism-related enzymes was observed (Table 5), suggesting that PEG and No-W affect carbon and proline metabolism through different mechanisms. 

An additional and very marked difference between both water-deficit treatments was the depletion of inorganic nutrients under PEG treatment (Figure 1 and Figure 2, Table S2). This phenomenon was also observed in wheat leaves subjected to PEG stress [41,42]. However, water withholding did not alter leaf ion content in *Limonium* species either [43], suggesting a lack of nutrient deficiency under these conditions. Maintaining appropriate nutrient levels is key for plant function and growth, and it is of special relevance under drought stress, where the decrease in transpiration and water uptake can lower K^+^ influx to the roots [44]. Considering the special importance of this nutrient, its decline, together with the decline in other essential nutrients suggest a higher plant metabolic and physiologic alteration and lower tolerance towards PEG treatment. These lower nutrient levels upon PEG exposure could be another consequence of low oxygen availability due to the highly viscous nature of the PEG solution, which could impair ion transport and uptake processes [33,45]. The presence of hypoxic conditions could also explain the vast increase in succinate and alanine levels in PEG-treated roots (Table 2 and Table 3) due to a shift in the primary carbon metabolism under these conditions [46].

### 3.2. Salinity and Drought: Differences in the Metabolic Response at the Whole-Plant Level

Although salt and drought stress trigger some common responses in plants, especially at early stages, salinity implies an important ionic stress by high intracellular sodium and chloride concentrations, which is absent in drought-stressed plants [47], thus affecting plant metabolism in a different way than no-irrigation stress.

The closure of stomata is a common phenomenon under water-deficit stress, aimed at decreasing water loss through transpiration and driven by a “hydroactive feedback” mechanism involving the leaf water status. This could explain why, despite the similar leaf water potential achieved by No-W and NaCl-stressed plants, drought stress led to a lower stomatal conductance, which correlated well with the lower leaf water content in the soil-drying experiment (Table 1, Figure 3). However, while salt stress did not significantly affect the roots’ water content, No-W conditions led to a significant decrease in this parameter (Table 1). This could mean that while the lack of irrigation led to an obvious decrease in the soil water content, the presence of NaCl and consequent increase in the soil osmotic potential was not enough to fully inhibit root water absorption under these conditions. Despite these physiological differences, it was at the metabolism level where the disparities between both treatments became more evident.

#### 3.2.1. Drought Stress Modulates Carbon and Antioxidant Metabolism in the Roots, While Salinity Does So in the Shoots

The differential response exerted by both water-deficit treatments was also visible when analyzing the carbohydrate content (Table 2, Figure 3). In leaves, the starch content increased in response to salt stress but decreased dramatically upon drought, while sucrose accumulated under both stresses, above all in response to NaCl, as observed in rice leaves [48]. The increase in leaf starch content is often observed in salinity experiments, and could be a means to scavenge excess sodium [37,49]. Additionally, this sugar partitioning into starch under NaCl stress could help avoid metabolic alterations derived by excessive accumulation of sucrose, which can act as a feedback inhibitor of processes such as photosynthesis [49]. On the other hand, soluble sugars, including fructose and, especially, sucrose and glucose, play a crucial role in plant metabolism and in the defense of plants against stress, serving as osmoprotectants, source of energy and carbon as well as signaling molecules involved in the up-regulation of genes involved in plant defense [39,49,50,51]. Hence, the accumulation of these molecules under abiotic stress has been widely reported in the literature [52].

In this study, phloem sucrose concentration was not altered under NaCl or No-W conditions (Table 2), suggesting that plant metabolism was not severely affected by these stresses, with enough starch available to sustain plant metabolism even at night [53]. Considering the fact that phloem translocation depends on source and sink strengths, the maintenance of the sucrose phloem transport despite the inhibition of photosynthesis under No-W conditions (Table 1), suggests an increased sink strength. This is in agreement with the significant increase in the root-to-shoot ratio under No-W (Table 1), a commonly observed phenomenon under drought stress linked to the lower growth inhibition of the root compared to the shoots, allowing better water uptake from the soil [54]. This increase in root strength under No-W could be linked to the increase in alkINV observed in this organ, which could compensate for the decrease in the SuSy activity (Table 5), as observed previously in arabidopsis [55]. The decrease in root SuSy activity in response to abiotic stress has been already reported for roots of salt-stressed maize [56] and drought-stressed sorghum [57] and *M. truncatula* [25], being described as one of the main and faster markers of abiotic stress [25]. In our study, the decrease in root sink strength due to a decrease in both SuSy and INV under salinity stress might indicate a redistribution of assimilates to the shoots rather than to the roots in NaCl-stressed *M. truncatula* plants. These responses suggest a key role of alkINV in the regulation of assimilate partitioning and plant response to drought stress, allowing sucrose transportation, unloading and its degradation in the root tissue for its use in root growth and/or osmoprotection under adverse conditions. 

In our study, the levels of leaf organic acids remained constant under No-W stress, while α-ketoglutarate and citrate increased significantly under salinity stress, reflecting the subtle differences in the management of the different osmotic stresses (Table 2, Figure 3)**.** The observed activation of IDH in salt-treated leaves (Table 5), in agreement with previous studies [58], not only could allow for this α-ketoglutarate (α-KG) build-up, but also for net glutamate biosynthesis via the GS/GOGAT cycle, thus playing a key role in the synthesis of amino acids as well as in the linkage between carbon and nitrogen metabolism [59]. In addition, the reaction catalyzed by IDH provides the cytosol with NADPH, which can be used in the reduction of NO_3_^−^ and many other biosynthetic processes such as proline biosynthesis or glutathione regeneration, allowing ROS scavenging [60]. This could explain why its over-expression increased salt tolerance in transgenic *A. thaliana* plants [61], hence suggesting an important role in *M. truncatula* tolerance to NaCl.

Interestingly, total glutathione and homoglutathione levels were lower in salt-stressed roots compared to those of No-W treated plants (Table 4, Figure 3). Glutathione is one of the main plant polar antioxidants and plays a central role in reactive oxygen species (ROS) scavenging through the glutathione–ascorbate cycle and as an electron donor to glutathione peroxidase, while homoglutathione is a glutathione homologue with similar functions and characteristic of legumes, usually being accumulated under various stresses, including drought [62]. The inherent ROS detoxifying properties of α-KG and its accumulation under NaCl stress together with the decreased ROS formation as a result of inhibited respiration due to lower NADH availability (through the potential inhibition of α-KGDH) confer a unique strategy to modulate the cellular redox environment [63]. Hence, the higher accumulation of α-KG in salt-treated leaves and drought-stressed-roots may reflect the higher need of these tissues for ROS detoxification. This apparently higher sensitivity of leaves to NaCl stress than roots was also reported for maize plants and switchgrass, with one of the responses being a higher antioxidant content in leaves than roots [64,65].

#### 3.2.2. Drought Elicits an Overall Accumulation of Amino Acids in Roots While Only Proline Metabolism Is Modulated under Salt Stress

Attending to nitrogen compounds, the response to salt and drought stress was also different (Table 3). Protein degradation is a common phenomenon observed under stress conditions contributing to the amino acid pool build-up [2]. In addition, the increased protein catabolism could also be linked to the provision of carbon sources for respiration [66]. In our study, soluble protein and total amino acid contents were not relevantly affected at either shoot or root level under salt stress. However, the lack of irrigation provoked an important accumulation of amino acids in the roots accompanied by a non-significant decrease in the protein content (Table 3) in agreement with previous studies [27,67]. In addition, the lack of phloem amino acid accumulation under drought stress opposed to the increase in leaves and roots suggests an in situ amino acid synthesis or protein recycling rather than import from the shoots. This is in agreement with the observed proteomic changes in *M. truncatula* [27], where drought stress provoked an accumulation of protein degradation and amino acid synthesis-related proteins in roots while decreasing in shoots, as suggested by our enzymatic studies (Table 5). In addition, the increased BCAA levels (Table S1), as previously observed [25], could be used as alternative electron donors for respiration, reinforcing their role on water-deficit tolerance [68].

Even though drought stress did not increase the phloem sap total amino acid content, Pro was significantly increased in agreement with studies in white clover [19] and arabidopsis [69]. Lee et al. concluded that Pro loading to the phloem sap has a significant influence on the down-regulation of N uptake and assimilation [19], which could imply a means of avoiding further ammonium build-up due to the down-regulation of growth and protein synthesis [70]. Indeed, the increased proline phloem loading in white clover was closely related to the decrease in nitrogen reductase in roots, nitrogen uptake and its assimilation [19].

Contrary to drought, salt stress did exert significant changes in the phloem sap amino acid content, with a significant increase in total and several individual amino acid contents, above all Pro and less significantly Gln, Ser, Ala and GABA (Table 3 and Table S1). The increase in total phloem sap amino acids was also observed in tomato plants [16], but the lack of increase in NaCl roots suggests their redistribution to older leaves, as previously observed for sucrose [71]. In addition, the increase in amino acid flow in the phloem could also be considered a nitrogen source, correlating well with the decrease in the NO_3_^−^ levels in the roots of NaCl plants (Figure 1), which further highlights the different response and adaptations of *M. truncatula* to both stresses. On the other hand, Cuin and Shabala suggested that certain amino acids such as GABA, Pro, Gln and Ser can mitigate the NaCl-induced K^+^ efflux, while others such as Val can enhance the detrimental effects of salinity on K^+^ homeostasis [72]. This is in agreement with the amino acid changes observed in the salt-stressed phloem sap, indicating that increases in free amino acids under abiotic stress might have a key role in plant salinity tolerance [72]. 

The overall accumulation of proline at the whole-plant level in response to both stresses was remarkable (Table 3), which is a widely accepted response of plants to abiotic stress. Proline is a compatible solute which acts as osmolyte, molecular chaperone and hydroxyl radical scavenger, protecting cells from stress-induced damage [2,73]. In our study, Pro synthesis was increased in both leaves and roots in NaCl-treated plants, while no-irrigation only altered Pro synthesis-related enzymes in the root tissue, correlating with Pro levels in each tissue (Table 3 and Table 5). On the contrary, Pro root degradation was only activated upon drought stress, possibly as a mechanism for energy and reducing equivalents generation under stressful conditions [74]. These results are in agreement with Verdoy et al., who observed an increased Pro degradation and activation of OAT activity in osmotic stressed *M. truncatula* roots, thus remarking the importance of the ornithine pathway under stressful conditions in this plant species [75]. 

#### 3.2.3. Salt Stress Provokes a Nutrient Deficiency Which Does Not Occur in Drought-Stressed Plants

The most notorious differential effect of both stresses was observed in the *M. truncatula* ionome (Figure 1 and Figure 3). Thus, while drought stress did not significantly alter the ion content of leaves and roots, salt stress led to massive ion alterations in both leaves and, above all, roots. NaCl irrigation led to a very important Na^+^ and Cl^−^ ion accumulation in both organs, as previously reported for alfalfa plants [76]. These ions’ transport to the shoot and vacuole sequestration is an extensively described salt tolerance mechanism for the maintenance of a healthy high K^+^/Na^+^ ratio [77]. The decrease in Ca^2+^ ions in the root tissue has also been previously observed in alfalfa plants under salinity stress [78], possibly related to a decreased uptake due to a reduced calcium activity in NaCl-rich soil solutions. Indeed, Na^+^ and Cl^−^ ions compete with other ions such as K^+^, Ca^2+^, Mg^2+^ and NH_4_^+^ as well as SO_4_^2−^ and NO_3_^−^, respectively, for the ion transporters during root uptake and translocation in plant shoots as well as during their use in biochemical processes such as enzymatic reactions [79].

The only significant effect of drought stress in the plant ionome was the accumulation of nitrate and, to a lesser extent, sulphate in the root tissue (Figure 1). Nitrate is the main nitrogen source in agricultural soils and the mineral nutrient that most frequently limits plant growth. This nitrate accumulation has been previously observed in *Spartina alterniflora* plants under drought stress [80] and could be due to a reduction in the nitrate reductase activity, the first and rate-limiting step enzyme in the nitrate assimilation pathway, which has been observed to decline in many species even under mild water-stress [81]. The decline in root AAT activity (Table 5), another nitrogen metabolism-related enzyme, also agrees with this decrease in root nitrogen metabolism under water-deficit conditions. However, despite the decrease in the primary assimilation of nitrogen, the increase in nitrogen compounds such as amino acids rules out a deficit in nitrogen under this stress.

## 4. Materials and Methods

### 4.1. Plant Materials and Stress Treatments

*Medicago truncatula* seeds were scarified with sulfuric acid 98% for 7 min, washed and then sterilized with 3.5% sodium hypochloride for 90 s. After a thorough wash, the seeds were soaked in water and left shaking in the dark for 6 h. When the seeds were hydrated, they were transferred to 7‰ agar plates at 4 °C for one day in the dark, and then incubated at 20 °C for two days. The seedlings were then planted in 1-L brown plastic pots with perlite:vermiculite (1:3, *v*/*v*) and grown for 11 weeks in a controlled chamber (22/18 °C day/night temperature, 70% relative humidity, 500 µmol m^−2^ s^−1^ (PPFD), 12-h photoperiod) and irrigated with Evans medium supplemented with 5 mM NH_4_NO_3_ [82].

NaCl, no-watering (No-W) and PEG-6000 stress were applied to provoke a drop of the leaf water potential (Ψ_leaf_) similar to a moderate water-deficit level. Salt stress was applied by watering plants with 0.25 M NaCl, which generated an osmolarity of 450 mmol Kg^−1^ in the solution. The No-W treatment was applied by limiting water irrigation to 2/3 of the daily-transpired water. Lastly, 250 g L^−1^ PEG-6000 were dissolved in water to reach the same osmolarity than the above-described salt solution. Control plants were watered daily with dH_2_O. All treatments were applied for 7 days. In addition to physiological measurements, different aliquots of leaf and lateral root tissue were collected and stored at −80 °C for further analysis. In the case of PEG-treated plants a large variation in the Ψ_leaf_ response was observed and only the ones with Ψ_leaf_ ≈ −1.7 MPa were selected for further experiments. 

### 4.2. Physiological Measurements

Plant transpiration was gravimetrically determined on a daily basis by weighting the pots one hour after the beginning of the photoperiod. Ψ_leaf_ was measured at this time point in the second fully expanded leaf using a pressure chamber (Soil Moisture Equipment, Santa Barbara, CA, USA) as earlier described [83]. Stomatal conductance was measured with a dynamic diffusion porometer (AP4; Delta-T devices, Cambridge, UK) in leaflets of the second-fully expanded leaf. Chlorophyll content was measured in five leaflets of the second-fully expanded leaf per plant using a SPAD-502 m (Konica-Minolta, Osaka, Japan). Water content (WC) was determined in base to the fresh weight (FW) and the dry weight (DW) obtained after 48 h drying at 70 °C using the following formula (1):

(1) $$\mathrm{WC}(\%)=[(100\times (\mathrm{FW}-\mathrm{DW})]{\left(\mathrm{FW}\right)}^{-1}$$

### 4.3. Phloem Sap Exudation

Phloem sap exudation was performed as indicated by Rahmat and Turnbull with minor modifications [84]. Petioles were cut with razor blades in buffer containing 10 mM EDTA, 10 mM HEPES pH 7. After gently drying the cut with paper, 10 leaves per plant were arranged and soaked in Eppendorf tubes containing 1.5 mL of the above-mentioned buffer. Phloem sap was exudated in the dark at 21 °C with the opening of the tube sealed with scotch tape and in saturated humidity conditions. After exudating for 22 h, the leaves were carefully removed from the tube and exudate extracts were frozen in liquid nitrogen and stored at −80 °C for further use.

### 4.4. PEG Uptake Measurement

The following procedure was adapted from the method described by Hyden [85]. Frozen leaves and roots aliquots (≈0.15 g) of all PEG-treated plants were homogenized in 1 mL extraction buffer (50 mM MOPS pH 7.5, 10 mM MgCl_2_, 20 mM KCl) and samples were centrifuged for 5 min at 11,500× *g*. The supernatant was then pipetted into a centrifuge tube, and 0.6 mL of 10% (*w*/*v*) BaCl·2H_2_0, 0.6 mL of saturated Ba(OH)_2_, and 0.6 mL of 5% (*w*/*v*) ZnSO_4_·7H_2_O were added with shaking between each addition. The tubes were allowed to stand for at least 5 min and then were centrifuged at 7600× *g*. The supernatant was mixed with 3 mL TCA solution (450 g TCA and 50 g BaCl_2_·2H_2_O in 1LH_2_O) and left for 5 min. The turbidity was then measured as the percentage of light absorbed at 600 nm, and the PEG content was calculated using a calibration curve [32]. The measured turbidity value in control plants was considered as zero.

### 4.5. Determination of Soluble Sugar and Starch Content

Frozen samples were extracted three times in boiling ethanol (80%, *v*/*v*) and once at RT for 30 s. The aqueous phase of the ethanol extract was evaporated at 40 °C and then the dry residue was resuspended in ddH_2_O by vigorous vortexing. A sample cleaning was then performed by centrifugation at 2330× *g* for 10 min and the supernatants were stored at −20 °C for further soluble sugar analysis. 

The remaining tissue after ethanol soluble compounds extraction was dried at 70 °C for 48 h. Dry samples were homogenized in ddH_2_O and further boiled for one hour for starch grain breakage. After cooling down, 1 mL of desalted amiloglucosidase solution (EC 3.2.1.3, 0.5 units/mL in acetate buffer pH 4.5) was added to each tube and incubated under shaking at 55 °C for starch digestion. Extractions were centrifuged at 2330× *g* for 10 min and the supernatants were stored at −20 °C for further glucose quantification. The soluble sugar analysis in the phloem sap was performed directly using the exudates. Sucrose, glucose and fructose were determined by capillary electrophoresis (CE) in a Coulter P/ACE system 5500 (Beckman Coulter Inc., Fullerton, CA, USA) coupled to a diode array detector [86]. Concentrations were calculated from peak heights using commercial standards (Sigma-Aldrich, Steinheim, Germany).

### 4.6. Determination of Organic Acids and Inorganic Ions

Frozen samples (≈100 mg) were homogenized to a fine powder in liquid N_2_ using a mortar and pestle. For organic and inorganic anions, 1.5 mL of 10% (*w*/*v*) TCA were added and the homogenate was centrifuged for 10 min at 1750× *g* and 4 °C. The aqueous phase was washed three times with ethyl ether saturated with water. The upper ether solution was discarded and the aqueous phase was purged with nitrogen gas for 2 min and subsequently filtered (0.45 nm pore size). For cations, finely-ground leaf and root material was extracted with 1 mL ddH_2_O for 30 min at 90 °C. After centrifugation (12,500× *g*, 10 min), the supernatant was collected and centrifuged again for 5 min. Ion levels were determined by ion chromatography in a DX-500 system (Dionex Ltd., Sunnvale, CA, USA). Cations were analyzed by isocratic separation (CS12A columns) and anions by gradient separation (AS11 column; 2.5 mM NaOH/18% methanol to 45 mM NaOH/18% methanol in 13 min) according to the manufacturer’s instructions.

### 4.7. Determination of Free Amino Acid Content

Frozen leaves (≈70 mg FW) and roots (≈150 mg FW) were ground to powder under liquid N_2_ and subsequently homogenized with 3 mL 1 M HCl. Homogenates were incubated on ice for 10 min and, subsequently, extracts were centrifuged twice at 20,000× *g* and 4 °C for 10 min. Supernatants were neutralized using NaOH, and internal standards norvaline and homoglutamic acid were spiked. In the case of the phloem sap, internal standards were directly added to the samples. Samples were then derivatized with 1 mM fluorescein isothiocyanate dissolved in acetone at RT for 15 h in 20 mM borate buffer pH 10. The content of free amino acids was determined using a Beckman Coulter capillary electrophoresis (CE) PA-800 (Beckman Coulter Inc., Brea, CA, USA) coupled to laser-induced fluorescence detection (argon laser at 488 nm), as described by Arlt et al. [87] and Takizawa and Nakamura [88], with minor modifications. 

A fused-silica capillary, 43/53.2 cm long and 50 µm internal diameter (Beckman Coulter Inc., Brea, CA, USA), was employed. For amino acid separation, 45 mM α-cyclodextrin in 80 mM borax buffer pH 9.2 was used. Analyses were performed at 20 °C and at a voltage of 30 kV (110 µA). Total amino acid content is presented as the summation of single amino acids for each sample and expressed on a DW basis.

### 4.8. Small Antioxidant Determination

Frozen samples (≈130 mg FW leaves and ≈180 mg FW roots) were ground to powder in liquid nitrogen and subsequently homogenized with 1 mL of ice-cold 2% metaphosphoric acid. The homogenate was centrifuged at 4400× *g* and 4 °C for 2 min. Antioxidants were analyzed by high-performance CE in a Beckman Coulter P/ACE system 5500 (Beckman coulter Inc., Fullerton, CA, USA.) associated with a diode array detector, as described by Herrero-Martínez et al. [89]. The CE instrument was equipped with the P/ACE station software for instrument control and data handling. The background buffer was 60 mM NaH_2_PO_4_ pH 7 containing 60 mM NaCl and 0.0001% hexadimethrine bromide. The applied potential was 15 kV, and the capillary tubing (50 μM) was 30/37 cm long. The indirect UV detection wavelength was set at 200 and 265 nm. Reduced ascorbate (ASC) glutathione (GSH) and homoglutathione (hGSH) were determined directly by injecting a plant extract aliquot in the CE as described above. Total ascorbate, glutathione and homoglutathione levels were directly analyzed by CE after sample reduction with DTT. Dehydroascorbate (DHA) and oxidized glutathione (GSSG) and homoglutathione (hGSSG) levels were determined as the difference between total ascorbate, glutathione and homoglutathione and their respective reduced form levels.

### 4.9. Enzymatic Activities and Total Soluble Protein Content

Leaf and root samples were ground into a fine powder with liquid nitrogen and homogenized with extraction buffer (50 mM MOPS pH 7.5, 0.1% (*v*/*v*) Triton X-100, 10 mM MgCl_2_, 1 mM EDTA, 20 mM KCl, 10 mM DTT, 10 mM β-mercaptoethanol, 2.5% (*w/v*) PVPP, 2 mM PMSF and a protease inhibition cocktail tablet) and centrifuged at 24,000× *g* and 4 °C for 20 min. The protein content of the supernatant was determined by the Bradford assay in the crude extract, while an aliquot was desalted through BioGel P-6 DG desalting gel for some enzymatic determinations. Enzymatic activities for aspartate aminotransferase (AAT), glutamate synthase (GOGAT), glutamine synthase (GS), glucose-6-phospate dehydrogenase (G6PDH), sucrose synthase (SuSy), NADP-isocitrate dehydrogenase (IDH), glutamate dehydrogenase (GDH), alkaline invertase (AlkINV), Δ^1^-1-pyrroline-5-carboxylate synthase (P5CS), ornithine aminotransferase (OAT) and proline dehydrogenase (ProDH) were performed as described previously [25].

### 4.10. Statistical Analysis

All data are reported as the mean ± standard error (SE) of 5 to 10 independent biological replicates. Multiple comparison analyses were performed with SPSS using analysis of variance (ANOVA, *p* < 0.05) and Tukey’s HSD test, while comparisons between treatment and control (when needed) were analyzed by Student’s *t*-test (*p* < 0.05). All mentioned correlations were statistically tested with XLSTAT 2020.5 (https://www.xlstat.com/en/, accessed on 23 December 2020) using Pearson’s correlation coefficient. Principal Component Analyses (PCA) were performed in log-transformed data using MetaboAnalyst 5.0 (https://www.metaboanalyst.ca/, accessed on 23 December 2020) and MetaGeneAlyse v1.7.1 (https://metagenealyse.mpimp-golm.mpg.de/, accessed on 23 December 2020).

## 5. Conclusions

**Figure 4:**
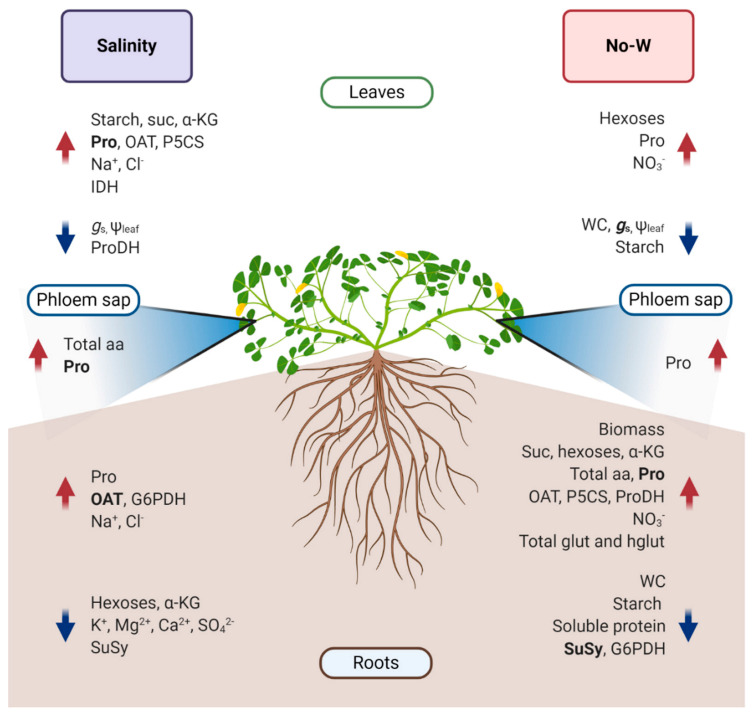
Summary of the most relevant *M. truncatula* responses to salinity and drought stress. This image depicts the increases and decreases in physiological parameters as well as enzymatic activities and metabolite and nutrient concentrations in leaves, roots and phloem sap under NaCl and no-irrigation (No-W) compared to control conditions. Bolded text indicates a higher increase or decrease in the studied parameter compared to the other stress. aa, amino acids; glut, glutathione; G6PDH, glucose-6-phosphate dehydrogenase; *g*_s_, stomatal conductance; hglut, homoglutathione; IDH, isocitrate dehydrogenase; α-KG, α-ketoglutarate; OAT, ornithine aminotransferase; P5CS, Δ^1^-1-pyrroline-5-carboxylate synthase; ProDH, proline dehydrogenase; Suc, sucrose; SuSy, sucrose synthase; WC, water content; Ψ_leaf_, leaf water potential. (Created with BioRender.com, accessed on 23 December 2020).

Generally, *M. truncatula* is considered a relatively drought tolerant legume compared to others like pea or soybean [90]. However, regarding salinity stress *M. truncatula* Jemalong A17 cultivar is considered salt sensitive by some authors [24] and salt tolerant by others [91]. Our study suggests a similar tolerance to both drought and salt stress, with even some parameters such as higher stomatal conductance and leaf water content suggesting slightly better coping with NaCl at similar leaf water potentials. Nonetheless, *M. truncatula* responses to both stresses differed notably (Figure 4), with a marked defensive response of NaCl shoots and droughted-roots through the accumulation of soluble sugars and amino acids under both stresses. In addition, the most relevant differences in the plant’s response to stress was the increased starch content in plants under NaCl, possibly as a mechanism of ion sequestration and avoidance of photosynthesis inhibition, as well as the increase in IDH activity and α-KG content. On the other hand, root proline catabolism, BCAA accumulation as well as the increased root sink strength via invertase could play key roles in *M. truncatula* response to no-irrigation stress. Regarding the use of PEG as a drought mimicker, even though the presence of typical drought stress markers such as proline accumulation could suggest PEG as an adequate drought simulator, the important impairment on nutrient uptake and the differential regulation of carbon and proline metabolism upon PEG treatment does not suggest that conclusions based on PEG studies may be directly extrapolated to the understanding of drought stress responses. Thus, results suggest a higher protection of shoots in salt stressed plants and roots in non-irrigated plants through different mechanisms including the regulation of proline and carbon metabolism, while discarding PEG as safe mimicker of drought. The conclusions derived from this work highlight the importance of the study of the response to abiotic stress in plants as whole systems and open the doors to future studies that research the relevance of the analyzed traits for water-deficit tolerance.

## Figures and Tables

**Table 1:** Physiological responses under different iso-osmotical conditions.

	Control	NaCl	No-W	PEG-6000
Total biomass (g DW)	16.7 ± 0.7 a	15.6 ± 1.0 a	17.0 ± 1.4 a	14.8 ± 0.9 a
Shoot biomass (g DW)	8.8 ± 0.3 a	8.4 ± 0.5 a	8.2 ± 0.6 a	7.82 ± 0.7 a
Root biomass (g DW)	7.5 ± 0.4 a	7.3 ± 0.4 a	8.8 ± 0.8 a	7.0 ± 0.3 a
Root-to-shoot ratio	0.81 ± 0.03 b	0.88 ± 0.03 b	1.07 ± 0.02 a	0.92 ± 0.08 ab
Leaf water potential (MPa)	−0.50 ± 0.00 a	−1.67 ± 0.02 b	−1.68 ± 0.02 b	−1.68 ± 0.02 b
Stomatal conductance (cm s^−1^)	0.087 ± 0.005 a	0.021 ± 0.003 b	0.000 ± 0.000 c	0.000 ± 0.000 c
Transpiration (g H_2_O day^−1^ plant^−1^)	174.1 ± 4.7 a	69.2 ± 8.7 b	50.5 ± 6.9 b	50.2 ± 5.0 b
Chlorophyll content (SPAD units)	64.0 ± 0.5 a	63.2 ± 1.0 a	63.4 ± 0.9 a	58.8 ± 0.4 b
Leaf water content (%)	81.7 ± 0.4 a	80.4 ± 0.8 ab	77.9 ± 0.7 b	68.6 ± 1.2 c
Root water content (%)	82.5 ± 0.8 a	80.2 ± 1.9 a	65.2 ± 1.8 b	83.5 ± 1.6 a

Values represent the means ± SE (*n* = 5–10). Letters represent statistical differences (Tukey’s test, *p* < 0.05) between treatments. No-W, no irrigation.

**Table 2:** Effect of the different water-stress treatments on the carbohydrate content.

		Control	NaCl	No-W	PEG-6000
Leaves	Starch	147.2 ± 29.1 b	330.1 ± 24.0 a	60.4 ± 16.2 c	0.000 ± 0.000 c
	Sucrose	11.5 ± 1.8 d	50.5 ± 4.9 b	25.6 ± 4.1 c	82.8 ± 16.6 ab
	Glucose	4.3 ± 1.0 b	6.6 ± 2.4 b	21.2 ± 5.4 a	9.1 ± 2.1 ab
	Fructose	6.1 ± 1.7 b	5.5 ± 1.5 b	15.5 ± 3.1 a	8.9 ± 2.2 ab
Phloem	Sucrose	17.8 ± 2.0 a	16.1 ± 3.8 a	18.0 ± 1.4 a	17.4 ± 1.6 a
Roots	Starch	10.4 ± 1.7 a	8.0 ± 2.1 ab	3.3 ± 0.4 b	6.0 ± 1.1 ab
	Sucrose	16.1 ± 1.8 c	28.0 ± 2.3 b	43.2 ± 1.9 a	45.3 ± 4.3 a
	Glucose	16.3 ± 2.6 b	4.3 ± 0.5 c	62.6 ± 9.8 a	8.2 ± 1.8 bc
	Fructose	26.8 ± 5.3 b	4.1 ± 0.8 c	74.4 ± 12.7 a	7.0 ± 1.4 c
Leaves	Malate	4.62 ± 1.45 a	5.61 ± 1.17 a	4.76 ± 0.39 a	2.90 ± 0.17 a
	Citrate	2.30 ± 0.93 ab	3.72 ± 0.65 a	1.04 ± 0.26 b	0.49 ± 0.05 b
	Succinate	0.12 ± 0.02 a	0.12 ± 0.01 a	0.11 ± 0.01 a	0.080 ± 0.006 a
	α-KG	0.040 ± 0.002 b	0.083 ± 0.006 a	0.031 ± 0.005 b	0.032 ± 0.002 b
Roots	Malate	1.21 ± 0.42 a	0.40 ± 0.06 a	1.51 ± 0.41 a	0.84 ± 0.09 a
	Citrate	0.59 ± 0.07 a	0.46 ± 0.10 a	0.47 ± 0.03 a	0.32 ± 0.02 a
	Succinate	0.095 ± 0.010 a	0.065 ± 0.005 ab	0.060 ± 0.008 b	0.55 ± 0.07 c
	α-KG	0.027 ± 0.003 a	0.012 ± 0.001 b	0.035 ± 0.002 a	0.030 ± 0.004 a

Effect of different treatments on the starch (measured as glucose units released after starch enzymatic breakdown) and various soluble sugars and organic acids (in µmol g dry weight (DW)^−1^) in leaf, root and phloem sap of *M. truncatula* plants. Values represent the means ± SE (*n* = 5). Letters represent statistical differences (Tukey’s test, *p* < 0.05) between treatments. α-KG, α-ketoglutarate; No-W, no-watering.

**Table 3:** Effect of the different water-stress treatments on the soluble protein and amino acid content.

		Control	NaCl	No-W	PEG-6000
**Leaf soluble protein**		150.3 ± 6.5 a	137.7 ± 4.5 a	140.4 ± 12.7 a	152.1 ± 15.9 a
**Root soluble protein**		27.8 ± 1.1 a	24.8 ± 1.4 ab	22.7 ± 1.2 ab	21.2 ± 1.6 b
**Leaves**	**Pro**	1.9 ± 0.1 d	24.4 ± 0.8 (13) b	16.1 ± 0.8 (9) c	56.7 ± 5.4 (30) a
	**aa**	85.2 ± 12.7 b	97.6 ± 14.8 b	118.6 ± 8.1 b	136.9 ± 8.5 (2) a
**Phloem**	**Pro**	0.17 ± 0.03 c	4.09 ± 0.68 (24) a	1.27 ± 0.13 (8) b	2.20 ± 0.48 (13) ab
	**aa**	8.8 ± 1.1 c	17.7 ± 1.7 (2) a	10.4 ± 0.7 b	17.6 ± 2.8 (2) a
**Roots**	**Pro**	0.47 ± 0.07 c	6.86 ± 1.20 (14) b	13.56 ± 0.92 (29) a	14.25 ± 1.21 (30) a
	**aa**	23.5 ± 4.3 c	30.3 ± 5.2 bc	78.0 ± 3.2 (3) a	63.1 ± 11.4 (3) ab

Response of *M. truncatula* leaves, phloem sap and roots to NaCl, no-watering (No-W) and polyethylene glycol (PEG) treatments regarding soluble protein (as mg g DW^−1^) and total soluble amino acid content (µmol g DW^−1^). Values represent the means ± SE (*n* = 5), while numbers in brackets represent the fold change of each treatment compared to control conditions when statistically significant according to *t*-Student’s test (*p* < 0.05). Letters represent statistical differences (Tukey’s test, *p* < 0.05) between treatments. aa, Total amino acids; Pro, Proline.

**Table 4:** Effect of the different water-stress treatments on the antioxidant composition of *M. truncatula*. As well as their reduced/total antioxidant content ratios in leaves and roots under control, NaCl, no watering (No-W) and PEG-6000 treatments.

		Control	NaCl	No-W	PEG
**Leaves**	**Total Asc**	3.41 ± 0.09 a	2.43 ± 0.08 b	3.00 ± 0.11 a	2.38 ± 0.11 b
	**ASC/Total Asc**	0.79 ± 0.06 a	0.74 ± 0.03 a	0.69 ± 0.02 a	0.63 ± 0.02 a
	**Total Glut**	0.70 ± 0.13 a	0.54 ± 0.03 a	0.67 ± 0.12 a	0.75 ± 0.07 a
	**GSH/Total Glut**	0.52 ± 0.03 a	0.44 ± 0.04 a	0.47 ± 0.01 a	0.30 ± 0.02 b
	**Total hGlut**	0.29 ± 0.03 a	0.31 ± 0.04 a	0.27 ± 0.06 a	0.18 ± 0.00 a
	**hGSH/Total hGlut**	0.44 ± 0.04 a	0.66 ± 0.07 a	0.51 ± 0.06 a	0.62 ± 0.08 a
**Roots**	**Total Asc**	0.58 ± 0.01 a	0.36 ± 0.05 b	0.23 ± 0.02 c	0.25 ± 0.03 bc
	**ASC/Total Asc**	0.36 ± 0.05 a	0.39 ± 0.01 a	n.d.	n.d.
	**Total Glut**	0.19 ± 0.02 b	0.17 ± 0.02 b	0.45 ± 0.04 a	0.21 ± 0.08 b
	**GSH/Total Glut**	0.65 ± 0.09 a	0.39 ± 0.04 a	0.36 ± 0.09 a	0.38 ± 0.09 a
	**Total hGlut**	0.52 ± 0.11 b	0.28 ± 0.03 b	1.23 ± 0.23 a	0.32 ± 0.06 b
	**hGSH/Total hGlut**	0.36 ± 0.06 a	0.42 ± 0.06 a	0.32 ± 0.04 a	0.38 ± 0.07 a

Values represent the means ± SE (*n* = 5). Letters represent statistical differences (Tukey’s test, *p* ≤ 0.05) between treatments. No reduced nor oxidized ascorbate data could be measured in the root tissue. Asc, ascorbate; ASC, reduced ascorbate; Glut, glutathione; *GSH*, reduced glutathione; hGlut, homoglutathione; hGSH, reduced homoglutathione; n.d., not detected.

**Table 5:** Effect of the different water-stress treatments on several enzymatic activities.

		Control	NaCl	No-W	PEG-6000
**Leaves**	**G6PDH**	0.12 ± 0.01 a	0.12 ± 0.02 a	0.18 ± 0.03 a	0.11 ± 0.03 a
	**IDH**	1.67 ± 0.10 b	2.38 ± 0.18 a	2.06 ± 0.31 ab	1.86 ± 0.14 ab
**Roots**	**AlkINV**	2.7 ± 0.2 ab	2.1 ± 0.5 b	3.7 ± 0.2 a	1.6 ± 0.4 b
	**G6PDH**	4.2 ± 0.1 b	6.1 ± 0.5 a	2.7 ± 0.1 b	2.6 ± 0.6 b
	**IDH**	7.6 ± 0.7 a	7.6 ± 0.6 a	6.3 ± 0.4 a	7.3 ± 1.2 a
	**SuSy**	2.7 ± 0.2 a	1.3 ± 0.3 b	1.0 ± 0.4 b	1.2 ± 0.5 ab
**Leaves**	**AAT**	3.3 ± 0.3 a	3.1 ± 0.4 a	3.2 ± 0.2 a	3.8 ± 0.3 a
	**GDH**	0.82 ± 0.06 a	0.83 ± 0.11 a	0.80 ± 0.04 a	0.85 ± 0.08 a
	**GOGAT**	0.063 ± 0.005 a	0.068 ± 0.014 a	0.040 ± 0.006 a	0.053 ± 0.009 a
	**GS**	0.027 ± 0.000 a	0.027 ± 0.001 a	0.030 ± 0.001 a	0.028 ± 0.003 a
**Roots**	**AAT**	15.1 ± 0.3 a	9.6 ± 1.3 b	7.7 ± 1.4 b	9.2 ± 1.2 b
	**GDH**	11.8 ± 1.5 a	16.8 ± 2.5 a	11.7 ± 1.6 a	13.0 ± 1.8 a
	**GOGAT**	0.25 ± 0.05 a	0.29 ± 0.06 a	0.20 ± 0.04 a	0.17 ± 0.03 a
**Leaves**	**OAT**	0.34 ± 0.04 a	0.55 ± 0.10 a	0.42 ± 0.03 a	0.15 ± 0.01 b
	**P5CS**	0.41 ± 0.03 b	0.59 ± 0.05 a	0.45 ± 0.03 ab	0.44 ± 0.02 ab
	**ProDH**	0.26 ± 0.03 a	0.17 ± 0.02 a	0.20 ± 0.01 a	0.24 ± 0.02 a
**Roots**	**OAT**	1.6 ± 0.1 b	2.6 ± 0.2 a	2.2 ± 0.1 ab	2.5 ± 0.2 a
	**P5CS**	0.64 ± 0.06 c	0.78 ± 0.09 bc	1.04 ± 0.09 ab	1.16 ± 0.05 a
	**ProDH**	0.52 ± 0.03 b	0.59 ± 0.03 b	0.91 ± 0.09 a	0.91 ± 0.05 a

Enzymatic activities of various carbon [alkaline invertase (alkINV), glucose-6-phosphate dehydrogenase (G6PDH), isocitrate dehydrogenase (IDH) and sucrose synthase (SuSy)], nitrogen [aspartate aminotransferase (AAT), glutamate dehydrogenase (GDH), glutamate synthase (GOGAT) and glutamine synthase (GS)] and proline metabolism [ornithine aminotransferase (OAT), Δ1-1-pyrroline-5-carboxylate synthase (P5CS) and proline dehydrogenase (ProDH)] (as nmol NADH min^−1^ µg prot^−1^) under control, NaCl, no-irrigation (No-W) and PEG stress in leaves and roots. Values represent the means ± SE (*n* = 5–10). Letters represent statistical differences (Tukey’s test, *p* < 0.05) between treatments.

## Data Availability

The data presented in this study are available in Table S5.

## Supplementary Materials

Supplementary materials can be found at https://www.mdpi.com/1422-0067/22/6/2813/s1. Figure S1: Visual effects in *M. truncatula* plants due to the different stress treatments.; Table S1: Absolute individual amino acid content in leaves, phloem sap and roots under different treatments; Table S2: Absolute individual inorganic ion content in leaves and roots under different treatments; Table S3: Antioxidant composition in leaves and roots under different treatments; Figure S2: Principal Component Analysis (PCA) considering all treatments; Table S4: Loading values resulting from the performed Principal Component Analyses (PCA); Table S5: All measurements performed in this study.

## Abbreviations

α-KG	α-Ketoglutarate
AAT	Aspartate aminotransferase
ABA	Abscisic acid
AlkINV	Alkaline invertase
ANOVA	Analysis of variance
ASC	Reduced ascorbate
Asc	Ascorbate
BCAA	Branched-chain amino acids
DHA	Dehydroascorbate
DW	Dry weight
FW	Fresh weight
G6PDH	Glucose-6-phosphate dehydrogenase
GABA	Gamma-aminobutyric acid
GDH	Glutamate dehydrogenase
Glut	Glutathione
g_s_	Stomatal conductance
GS/GOGAT	Glutamine synthetase/Glutamine oxoglutarate aminotransferase
GSH	Reduced glutathione
GSSG	Oxidized glutathione
hGlut	Homoglutathione
hGSH	Reduced homoglutathione
hGSSG	Oxidized homoglutathione
IDH	NADP-isocitrate dehydrogenase
No-W	No watering
OAT	Ornithine-delta-aminotransferase
P5CS	Pyrroline-5-carboxylate synthase
PCA	Principal Component Analysis
PEG	Polyethylene glycol
ProDH	Proline dehydrogenase
ROS	Reactive oxygen species
SuSy	Sucrose synthase
WC	Water content

## References

[1] Mahajan S., Tuteja N. (2005). Cold, salinity and drought stresses: An overview. Arch. Biochem. Biophys..

[2] Krasensky J., Jonak C. (2012). Drought, salt, and temperature stress-induced metabolic rearrangements and regulatory networks. J. Exp. Bot..

[3] Larrainzar E., Molenaar J.A., Wienkoop S., Gil-Quintana E., Alibert B., Limami A., Arrese-Igor C., González E.M. (2014). Drought stress provokes the down-regulation of methionine and ethylene biosynthesis pathways in *Medicago truncatula* roots and nodules. Plant. Cell Environ..

[4] Nunes C.C., de Sousa Araújo S., da Silva J.M., Fevereiro M.P.S., da Silva A.B. (2008). Physiological responses of the legume model *Medicago truncatula* cv. Jemalong to water deficit. Environ. Exp. Bot..

[5] Miao Z., Xu W., Li D., Hu X., Liu J., Zhang R., Tong Z., Dong J., Su Z., Zhang L. (2015). De novo transcriptome analysis of *Medicago falcata* reveals novel insights about the mechanisms underlying abiotic stress-responsive pathway. BMC Genom..

[6] Do P.T., Drechsel O., Heyer A.G., Hincha D.K., Zuther E. (2014). Changes in free polyamine levels, expression of polyamine biosynthesis genes, and performance of rice cultivars under salt stress: A comparison with responses to drought. Front. Plant Sci..

[7] Staudinger C., Mehmeti V., Turetschek R., Lyon D., Egelhofer V., Wienkoop S. (2012). Possible role of nutritional priming for early salt and drought stress responses in *Medicago truncatula*. Front. Plant Sci..

[8] Cui G., Zhang Y., Zhang W., Lang D., Zhang X., Li Z., Zhang X. (2019). Response of carbon and nitrogen metabolism and secondary metabolites to drought stress and salt stress in plants. J. Plant Biol..

[9] Nunes-Nesi A., Fernie A.R., Stitt M. (2010). Metabolic and signaling aspects underpinning the regulation of plant carbon nitrogen interactions. Mol. Plant.

[10] Griffiths C.A., Paul M.J., Foyer C.H. (2016). Metabolite transport and associated sugar signalling systems underpinning source/sink interactions. Biochim. Biophys. Acta.

[11] Lemoine R., La Camera S., Atanassova R., Dédaldéchamp F., Allario T., Pourtau N., Bonnemain J.-L., Laloi M., Coutos-Thévenot P., Maurousset L. (2013). Source-to-sink transport of sugar and regulation by environmental factors. Front. Plant Sci..

[12] Sevanto S. (2014). Phloem transport and drought. J. Exp. Bot..

[13] Savage J.A., Clearwater M.J., Haines D.F., Klein T., Mencuccini M., Sevanto S., Turgeon R., Zhang C. (2016). Allocation, stress tolerance and carbon transport in plants: How does phloem physiology affect plant ecology?. Plant Cell Environ..

[14] Sevanto S. (2018). Drought impacts on phloem transport. Curr. Opin. Plant Biol..

[15] Castañeda V., González E.M., Wienkoop S. (2021). Phloem sap proteins are part of a core stress responsive proteome involved in drought stress adjustment. Front. Plant Sci..

[16] Pérez-Alfocea F., Balibrea M.E., Alarcón J.J., Bolarín M.C. (2000). Composition of xylem and phloem exudates in relation to the salt-tolerance of domestic and wild tomato species. J. Plant Physiol..

[17] Ogden A.J., Bhatt J.J., Brewer H.M., Kintigh J., Kariuki S.M., Rudrabhatla S., Adkins J.N., Curtis W.R. (2020). Phloem exudate protein profiles during drought and recovery reveal abiotic stress responses in tomato vasculature. Int. J. Mol. Sci..

[18] Pommerrenig B., Papini-Terzi F.S., Sauer N. (2007). Differential regulation of sorbitol and sucrose loading into the phloem of *Plantago major* in response to salt stress. Plant Physiol..

[19] Lee B.R., Jin Y.L., Avice J.C., Cliquet J.B., Ourry A., Kim T.H. (2009). Increased proline loading to phloem and its effects on nitrogen uptake and assimilation in water-stressed white clover (*Trifolium repens*). New Phytol..

[20] Jeschke W., Pate J. (1991). Cation and chloride partitioning through xylem and phloem within the whole plant of *Ricinus communis* L. under conditions of salt stress. J. Exp. Bot..

[21] Lohaus G., Hussmann M., Pennewiss K., Schneider H., Zhu J.J., Sattelmacher B. (2000). Solute balance of a maize (*Zea mays* L.) source leaf as affected by salt treatment with special emphasis on phloem retranslocation and ion leaching. J. Exp. Bot..

[22] Girousse C., Bournoville R., Bonnemain J.L. (1996). Water deficit-induced changes in concentrations in proline and some other amino acids in the phloem sap of alfalfa. Plant Physiol..

[23] Luo S.S., Sun Y.N., Zhou X., Zhu T., Zhu L.S., Arfan M., Zou L.J., Lin H.H. (2016). *Medicago truncatula* genotypes Jemalong A17 and R108 show contrasting variations under drought stress. Plant Physiol. Biochem..

[24] Long R., Li M., Zhang T., Kang J., Sun Y., Cong L., Gao Y., Liu F., Yang Q. (2016). Comparative proteomic analysis reveals differential root proteins in *Medicago sativa* and *Medicago truncatula* in response to salt stress. Front. Plant Sci..

[25] Castañeda V., de la Peña M., Azcárate L., Aranjuelo I., González E. (2019). Functional analysis of the taproot and fibrous roots of *Medicago truncatula*: Sucrose and proline catabolism primary response to water deficit. Agric. Water Manag..

[26] Zhang J.Y.Y., Cruz de Carvalho M.H., Torres-Jerez I., Kang Y., Allen S.N., Huhman D.V., Tang Y., Murray J., Sumner L.W., Udvardi M.K. (2014). Global reprogramming of transcription and metabolism in *Medicago truncatula* during progressive drought and after rewatering. Plant Cell Environ..

[27] Lyon D., Castillejo M.A., Mehmeti-Tershani V., Staudinger C., Kleemaier C., Wienkoop S. (2016). Drought and recovery: Independently regulated processes highlighting the importance of protein turnover dynamics and translational regulation in *Medicago truncatula*. Mol. Cell. Proteom..

[28] Dubois M., Inzé D. (2020). Plant growth under suboptimal water conditions: Early responses and methods to study them. J. Exp. Bot..

[29] Jacomini E., Bertani A., Mapelli S. (1988). Accumulation of polyethylene glycol 6000 and its effects on water content and carbohydrate level in water-stressed tomato plants. Can. J. Bot..

[30] Yaniv Z., Werker E. (1983). Absorption and secretion of polyethylene glycol by *Solanaceous* plants. J. Exp. Bot..

[31] Lawlor D.W. (1970). Absorption of polyethylene glycols by plants and their effects on plant growth. New Phytol..

[32] Janes B.E. (1974). The effect of molecular size, concentration in nutrient solution, and exposure time on the amount and distribution of polyethylene glycol in pepper plants. Plant Physiol..

[33] Osmolovskaya N., Shumilina J., Kim A., Didio A., Grishina T., Bilova T., Keltsieva O.A., Zhukov V., Tikhonovich I., Tarakhovskaya E. (2018). Methodology of drought stress research: Experimental setup and physiological characterization. Int. J. Mol. Sci..

[34] Fang Y., Xiong L. (2014). General mechanisms of drought response and their application in drought resistance improvement in plants. Cell. Mol. Life Sci..

[35] Fan S., Blake T.J. (1997). Comparison of polyethylene glycol 3350 induced osmotic stress and soil drying for drought simulation in three woody species. Trees.

[36] Chazen O., Hartung W., Neumann P.M. (1995). The different effects of PEG-6000 and NaCl on leaf development are associated with differential inhibition of root water transport. Plant Cell Environ..

[37] Thalmann M., Santelia D. (2017). Starch as a determinant of plant fitness under abiotic stress. New Phytol..

[38] Darko E., Végh B., Khalil R., Marček T., Szalai G., Pál M., Janda T. (2019). Metabolic responses of wheat seedlings to osmotic stress induced by various osmolytes under iso-osmotic conditions. PLoS ONE.

[39] Rosa M., Prado C., Podazza G., Interdonato R., González J.A., Hilal M., Prado F.E. (2009). Soluble sugars: Metabolism, sensing and abiotic stress: A complex network in the life of plants. Plant Signal. Behav..

[40] Cui G., Zhao Y., Zhang J., Chao M., Xie K., Zhang C., Sun F., Liu S., Xi Y. (2019). Proteomic analysis of the similarities and differences of soil drought and polyethylene glycol stress responses in wheat (*Triticum aestivum* L.). Plant Mol. Biol..

[41] Pei Z.F., Ming D.F., Liu D., Wan G.L., Geng X.X., Gong H.J., Zhou W.J. (2010). Silicon improves the tolerance to water-deficit stress induced by polyethylene glycol in wheat (*Triticum aestivum* L.) seedlings. J. Plant Growth Regul..

[42] Filek M., Walas S., Mrowiec H., Rudolphy-Skórska E., Sieprawska A., Biesaga-Kościelniak J. (2012). Membrane permeability and micro- and macroelement accumulation in spring wheat cultivars during the short-term effect of salinity- and PEG-induced water stress. Acta Physiol. Plant..

[43] González-Orenga S., Al Hassan M., Llinares J.V., Lisón P., López-Gresa M.P., Verdeguer M., Vicente O., Boscaiu M. (2019). Qualitative and quantitative differences in osmolytes accumulation and antioxidant activities in response to water deficit in four mediterranean *Limonium* species. Plants.

[44] Wang M., Zheng Q., Shen Q., Guo S. (2013). The critical role of potassium in plant stress response. Int. J. Mol. Sci..

[45] Munns R., James R.A., Sirault X.R.R., Furbank R.T., Jones H.G. (2010). New phenotyping methods for screening wheat and barley for beneficial responses to water deficit. J. Exp. Bot..

[46] António C., Päpke C., Rocha M., Diab H., Limami A., Obata T., Fernie A., van Dongen J. (2016). Regulation of primary metabolism in response to low oxygen availability as revealed by carbon and nitrogen isotope redistribution. Plant Physiol.

[47] Munns R. (2011). Plant adaptations to salt and water stress: Differences and commonalities. Adv. Bot. Res..

[48] Amirjani M.R. (2011). Effect of salinity stress on growth, sugar content, pigments and enzyme activity of rice. Int. J. Bot..

[49] Dong S., Beckles D.M. (2019). Dynamic changes in the starch-sugar interconversion within plant source and sink tissues promote a better abiotic stress response. J. Plant Physiol..

[50] Gibson S.I. (2000). Plant sugar-response pathways. Part of a complex regulatory web. Plant Physiol..

[51] Ruan Y.L., Jin Y., Yang Y.J., Li G.J., Boyer J.S. (2010). Sugar input, metabolism, and signaling mediated by invertase: Roles in development, yield potential, and response to drought and heat. Mol. Plant.

[52] Singh M., Kumar J., Singh S., Singh V.P., Prasad S.M. (2015). Roles of osmoprotectants in improving salinity and drought tolerance in plants: A review. Rev. Environ. Sci. Bio/Technol..

[53] Dong S., Zhang J., Beckles D.M. (2018). A pivotal role for starch in the reconfiguration of ^14^C-partitioning and allocation in *Arabidopsis thaliana* under short-term abiotic stress. Sci. Rep..

[54] Gargallo-Garriga A., Sardans J., Pérez-Trujillo M., Rivas-Ubach A., Oravec M., Vecerova K., Urban O., Jentsch A., Kreyling J., Beierkuhnlein C. (2014). Opposite metabolic responses of shoots and roots to drought. Sci. Rep..

[55] Barratt D.H.P., Derbyshire P., Findlay K., Pike M., Wellner N., Lunn J., Feil R., Simpson C., Maule A.J., Smith A.M. (2009). Normal growth of arabidopsis requires cytosolic invertase but not sucrose synthase. Proc. Natl. Acad. Sci. USA.

[56] Hütsch B.W., Osthushenrich T., Faust F., Kumar A., Schubert S. (2016). Reduced sink activity in growing shoot tissues of maize under salt stress of the first phase may be compensated by increased PEP-carboxylase activity. J. Agron. Crop. Sci..

[57] Goche T., Shargie N.G., Cummins I., Brown A.P., Chivasa S., Ngara R. (2020). Comparative physiological and root proteome analyses of two sorghum varieties responding to water limitation. Sci. Rep..

[58] Naliwajski M.R., Skłodowska M. (2018). The relationship between carbon and nitrogen metabolism in cucumber leaves acclimated to salt stress. PeerJ.

[59] Foyer C.H., Noctor G., Hodges M. (2011). Respiration and nitrogen assimilation: Targeting mitochondria-associated metabolism as a means to enhance nitrogen use efficiency. J. Exp. Bot..

[60] Corpas F.J., Barroso J.B. (2014). NADPH-generating dehydrogenases: Their role in the mechanism of protection against nitro-oxidative stress induced by adverse environmental conditions. Front. Environ. Sci..

[61] Liu Y., Shi Y., Song Y., Wang T., Li Y. (2010). Characterization of a stress-induced NADP^+^-isocitrate dehydrogenase gene in maize confers salt tolerance in arabidopsis. J. Plant Biol..

[62] Zagorchev L., Seal C., Kranner I., Odjakova M. (2013). A central role for thiols in plant tolerance to abiotic stress. Int. J. Mol. Sci..

[63] Mailloux R.J., Bériault R., Lemire J., Singh R., Chénier D.R., Hamel R.D., Appanna V.D. (2007). The tricarboxylic acid cycle, an ancient metabolic network with a novel twist. PLoS ONE.

[64] Qing D.J., Lu H.F., Li N., Dong H.T., Dong D.F., Li Y.Z. (2009). Comparative profiles of gene expression in leaves and roots of maize seedlings under conditions of salt stress and the removal of salt stress. Plant Cell Physiol..

[65] Wang Q., Wu C., Xie B., Liu Y., Cui J., Chen G., Zhang Y. (2012). Model analysing the antioxidant responses of leaves and roots of switchgrass to NaCl-salinity stress. Plant Physiol. Biochem..

[66] Araújo W.L., Tohge T., Ishizaki K., Leaver C.J., Fernie A.R. (2011). Protein degradation—An alternative respiratory substrate for stressed plants. Trends Plant Sci..

[67] Akhzari D., Pessarakli M. (2016). Effect of drought stress on total protein, essential oil content, and physiological traits of *Levisticum officinale* Koch. J. Plant Nutr..

[68] Pires M., Pereira A.A., Medeiros D.B., Daloso D.M., Pham P.A., Barros K.A., Engqvist M.K.M., Florian A., Krahnert I., Maurino V.G. (2016). The influence of alternative pathways of respiration that utilize branched-chain amino acids following water shortage in arabidopsis. Plant Cell Environ..

[69] Mewis I., Khan M.A.M., Glawischnig E., Schreiner M., Ulrichs C. (2012). Water stress and aphid feeding differentially influence metabolite composition in *Arabidopsis thaliana* (L.). PLoS ONE.

[70] Kim T.H., Lee B.R., Jung W.J., Kim K.Y., Avice J.C., Ourry A. (2004). *De novo* protein synthesis in relation to ammonia and proline accumulation in water stressed white clover. Funct. Plant Biol..

[71] Munns R., Tester M. (2008). Mechanisms of salinity tolerance. Annu. Rev. Plant Biol..

[72] Cuin T.A., Shabala S. (2007). Amino acids regulate salinity-induced potassium efflux in barley root epidermis. Planta.

[73] Sharma S., Villamor J.G., Verslues P.E. (2011). Essential role of tissue-specific proline synthesis and catabolism in growth and redox balance at low water potential. Plant Physiol..

[74] Kaur G., Asthir B. (2015). Proline: A key player in plant abiotic stress tolerance. Biol. Plant..

[75] Verdoy D., Coba de la Pena T., Redondo F.J., Lucas M.M., Pueyo J.J. (2006). Transgenic *Medicago truncatula* plants that accumulate proline display nitrogen-fixing activity with enhanced tolerance to osmotic stress. Plant Cell Environ..

[76] Sun J., Yang G., Zhang W., Zhang Y. (2016). Effects of heterogeneous salinity on growth, water uptake, and tissue ion concentrations of alfalfa. Plant Soil.

[77] Nadeem M., Li J., Yahya M., Wang M., Ali A., Cheng A., Wang X., Ma C. (2019). Grain legumes and fear of salt stress: Focus on mechanisms and management strategies. Int. J. Mol. Sci..

[78] Ashrafi E., Razmjoo J., Zahedi M. (2018). Effect of salt stress on growth and ion accumulation of alfalfa (*Medicago sativa* L.) cultivars. J. Plant Nutr..

[79] Parihar P., Singh S., Singh R., Singh V.P., Prasad S.M. (2015). Effect of salinity stress on plants and its tolerance strategies: A review. Environ. Sci. Pollut. Res..

[80] Hessini K., Martínez J.P., Gandour M., Albouchi A., Soltani A., Abdelly C. (2009). Effect of water stress on growth, osmotic adjustment, cell wall elasticity and water-use efficiency in *Spartina alterniflora*. Environ. Exp. Bot..

[81] Correia M.J., Fonseca F., Azedo-Silva J., Dias C., David M.M., Barrote I., Osorio M.L., Osorio J. (2005). Effects of water deficit on the activity of nitrate reductase and content of sugars, nitrate and free amino acids in the leaves and roots of sunflower and white lupin plants growing under two nutrient supply regimes. Physiol. Plant..

[82] Evans H.J., Moore T. (1981). Symbiotic nitrogen fixation in legume nodules. Research Experiences in Plant Physiology.

[83] Scholander P.F., Bradstreet E.D., Hemmingsen E.A., Hammel H.T. (1965). Sap pressure in vascular plants: Negative hydrostatic pressure can be measured in plants. Science.

[84] Rahmat Z., Turnbull C. (2013). Optimization of EDTA edta exudation technique for proteome study of the phloem. J. Teknol..

[85] Hyden S. (1956). A turbidimetric method for the determination of higher polyethylene glycols in biological materials. K. Lantbr. Ann..

[86] Marino D., González E.M., Arrese-Igor C. (2006). Drought effects on carbon and nitrogen metabolism of pea nodules can be mimicked by paraquat: Evidence for the occurrence of two regulation pathways under oxidative stresses. J. Exp. Bot..

[87] Arlt K., Brandt S., Kehr J. (2001). Amino acid analysis in five pooled single plant cell samples using capillary electrophoresis coupled to laser-induced fluorescence detection. J. Chromatogr. A.

[88] Takizawa K., Nakamura H. (1998). Separation and determination of fluorescein isothiocyanate-labeled amino acids by capillary electrophoresis with laser-induced fluorescence detection. Anal. Sci..

[89] Herrero-Martínez J., Simó-Alfonso E.F., Ramis-Ramos G., Deltoro V.I., Calatayud A., Barreno E. (2000). Simultaneous determination of L-ascorbic acid, glutathione, and their oxidized forms in ozone-exposed vascular plants by capillary zone electrophoresis. Environ. Sci. Technol..

[90] Limami A., Ricoult C., Planchet E., González E., Ladrera R., Larrainzar E., Arrese-Igor C., Merchan F., Crespi M., Frugier F., Mathesius U., Journet E., Sumner L. (2007). Response of *Medicago truncatula* to abiotic stress. Medicago Truncatula Handbook.

[91] De Bruijn F.J. (2020). Genetic map of *Medicago truncatula*. The Model Legume Medicago Truncatula.

