# Supplementary material for: Strategies to Apply Water-Deficit Stress: Similarities and Disparities at the Whole Plant Metabolism Level in Medicago truncatula
Source: Int J Mol Sci. 2021 Mar 10;22(6):2813. doi: 10.3390/ijms22062813 (PMC8002188; doi:10.3390/ijms22062813)
Supplement: Supplementary file 1 [file ijms-22-02813-s001.zip › ijms-1103292-revision-suppl/Figure S1.pdf]

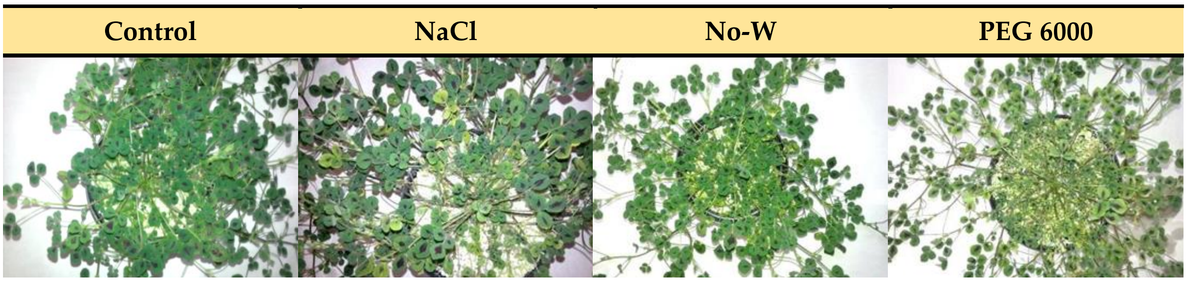

Figure S1. Visual effects in *M. truncatula plants* due to the different stress treatments. Twelve-week-old *Medicago truncatula* plants were subjected to iso-osmotical conditions of salinity and osmotic stress. The addition of 0.25 M NaCl lead to a slight yellowing of the older leaves. No-Watering (No-W) treatment consisted of limiting water irrigation to 2/3 of the daily transpired water and did not provoke noticeable changes in the shoot, except for a slight turgor loss. On the other hand, the addition of 250 g l^{-1} PEG 6000 to the irrigation solution lead to a less leafy appearance and the slight whitening of the leaves.
