# Supplementary material for: Strategies to Apply Water-Deficit Stress: Similarities and Disparities at the Whole Plant Metabolism Level in Medicago truncatula
Source: Int J Mol Sci. 2021 Mar 10;22(6):2813. doi: 10.3390/ijms22062813 (PMC8002188; doi:10.3390/ijms22062813)
Supplement: Supplementary file 1 [file ijms-22-02813-s001.zip › ijms-1103292-revision-suppl/Table S1.pdf]

Table S1. Absolute individual amino acid content in leaves, phloem sap and roots under different water-deficit treatments. Response of *M. truncatula* leaves, phloem sap and roots to NaCl, no-watering (No-W) and PEG treatments regarding soluble amino acid content (μmol g DW^{-1}). Values represent the means ± SE (n = 5), while numbers in brackets represent the fold change of each treatment compared to control conditions when statistically significant according to t-Student's test (P <0.05). Letters represent statistical differences (Tukey`s test, P <0.05) between treatments.

		Control		NaCl			No-W			PEG 6000		
Leaf soluble amino acid content	Arg	0.68 ± 0.17	ab	0.31 ± 0.06		b	0.87 ± 0.12		a	1.17 ± 0.22		a
	Lys	0.55 ± 0.10	b	0.61 ± 0.06		b	1.24 ± 0.13	(2)	a	0.90 ± 0.08	(2)	ab
	Leu	0.83 ± 0.09	b	0.62 ± 0.10		b	1.41 ± 0.05	(2)	a	0.82 ± 0.10		b
	Ile	0.79 ± 0.15	b	0.81 ± 0.11		b	1.70 ± 0.06	(2)	a	1.04 ± 0.12		b
	Met	0.13 ± 0.01	b	0.15 ± 0.02		b	0.31 ± 0.02	(2)	a	0.22 ± 0.03		ab
	Phe	0.80 ± 0.09	c	1.10 ± 0.24		bc	2.08 ± 0.12	(3)	ab	2.94 ± 0.32	(4)	a
	Trp	0.50 ± 0.06	c	0.88 ± 0.17		bc	1.38 ± 0.11	(3)	ab	1.74 ± 0.20	(3)	a
	His	0.60 ± 0.02	c	0.92 ± 0.18		bc	1.33 ± 0.16	(2)	ab	2.39 ± 0.33	(4)	a
	Tyr	0.51 ± 0.06	ab	0.37 ± 0.04		b	0.72 ± 0.06		a	0.60 ± 0.07		ab
	Val	2.2 ± 0.4	b	2.2 ± 0.3		b	5.0 ± 0.2	(2)	a	5.1 ± 0.3	(2)	a
	Gln	0.81 ± 0.04	ab	0.56 ± 0.11		b	1.41 ± 0.10	(2)	a	1.24 ± 0.30		ab
	Pro	1.9 ± 0.1	d	24.4 ± 0.8	(13)	b	16.1 ± 0.8	(9)	c	56.7 ± 5.4	(30)	a
	Asn	48.3 ± 11.4	a	30.2 ± 10.5		a	57.2 ± 6.8		a	42.4 ± 4.3		a
	GABA	0.65 ± 0.13	a	0.48 ± 0.09		a	0.73 ± 0.07		a	0.74 ± 0.08		a
	Thr	2.56 ± 0.19	b	2.02 ± 0.24		bc	3.78 ± 0.18		a	1.36 ± 0.07	(-2)	c
	Ser	4.6 ± 1.2	b	15.3 ± 3.6	(3)	a	3.6 ± 0.6		b	5.7 ± 0.4		b
	Gly	0.29 ± 0.03	b	0.28 ± 0.03		b	0.42 ± 0.03		ab	0.50 ± 0.04	(2)	a
	Ala	2.1 ± 0.4	a	2.2 ± 0.5		a	2.6 ± 0.2		a	2.0 ± 0.1		a
	Glu	11.3 ± 1.5	ab	10.7 ± 1.7		ab	13.0 ± 0.9		a	7.4 ± 0.5		b
	Asp	5.1 ± 0.7	a	3.4 ± 0.5		ab	3.7 ± 0.1		a	2.0 ± 0.1	(-3)	b
	**TOTAL**	**85.2 ± 12.7**	**b**	**97.6 ± 14.8**		**b**	**118.6 ± 8.1**		**b**	**136.9 ± 8.5**	**(2)**	**a**
Phloem sap soluble amino acid content	Arg	0.055 ± 0.006	b	0.12 ± 0.01		a	0.048 ± 0.008		b	0.075 ± 0.019		ab
	Lys	0.30 ± 0.03	a	0.23 ± 0.02		a	0.23 ± 0.02		a	0.19 ± 0.04		a
	Leu	0.17 ± 0.03	a	0.15 ± 0.02		ab	0.078 ± 0.003	(-2)	c	0.094 ± 0.020		bc
	Ile	0.23 ± 0.04	a	0.12 ± 0.01		b	0.14 ± 0.00		ab	0.10 ± 0.02	(-2)	b
	Met	0.016 ± 0.003	a	0.023 ± 0.004		a	0.014 ± 0.002		a	0.017 ± 0.004		a
	Phe	0.10 ± 0.02	a	0.087 ± 0.006		a	0.073 ± 0.006		a	0.087 ± 0.019		a
	Trp	0.024 ± 0.006	a	0.010 ± 0.003		a	0.013 ± 0.003		a	0.014 ± 0.002		a
	His	0.10 ± 0.02	a	0.061 ± 0.010		a	0.058 ± 0.009		a	0.084 ± 0.023		a
	Tyr	0.083 ± 0.009	ab	0.099 ± 0.010		a	0.056 ± 0.004		b	0.098 ± 0.017		ab
	Val	0.66 ± 0.08	a	0.36 ± 0.04	(-2)	b	0.53 ± 0.01		a	0.49 ± 0.05		ab
	Gln	0.14 ± 0.03	c	0.68 ± 0.14	(5)	a	0.17 ± 0.01		bc	0.38 ± 0.03	(3)	ab
	Pro	0.17 ± 0.03	c	4.09 ± 0.68	(24)	a	1.27 ± 0.13	(8)	b	2.20 ± 0.48	(13)	ab
	Asn	3.8 ± 0.7	b	5.4 ± 1.0		ab	3.5 ± 0.3		b	7.9 ± 1.2	(2)	a
	GABA	0.59 ± 0.06	c	1.86 ± 0.29	(3)	a	0.72 ± 0.05		c	1.42 ± 0.23	(2)	b
	Thr	0.24 ± 0.04	a	0.34 ± 0.04		a	0.21 ± 0.01		a	0.30 ± 0.05		a

	**Ser**	0.55 ± 0.15	b	1.27 ± 0.20	(2)	a	0.34 ± 0.06		b	0.54 ± 0.07		b
	**Gly**	0.048 ± 0.008	a	0.073 ± 0.009		a	0.047 ± 0.005		a	0.067 ± 0.007		a
	**Ala**	0.25 ± 0.02	b	0.50 ± 0.08	(2)	a	0.29 ± 0.03		ab	0.38 ± 0.07		ab
	**Glu**	0.42 ± 0.05	a	0.74 ± 0.13		a	0.70 ± 0.12		a	0.72 ± 0.16		a
	**Asp**	0.79 ± 0.19	b	1.46 ± 0.41		ab	1.91 ± 0.20	(2)	a	2.51 ± 0.62	(3)	a
	**TOTAL**	**8.8 ± 1.1**	**c**	**17.7 ± 1.7**	**(2)**	**a**	**10.4 ± 0.7**		**b**	**17.6 ± 2.8**	**(2)**	**a**
**Root soluble amino acid content**	**Arg**	0.13 ± 0.02	b	0.21 ± 0.05		ab	0.41 ± 0.04	(3)	a	0.43 ± 0.09	(3)	a
	**Lys**	0.22 ± 0.05	b	0.27 ± 0.04		b	0.69 ± 0.06	(3)	a	0.55 ± 0.15		ab
	**Leu**	0.22 ± 0.03	b	0.19 ± 0.02		b	0.52 ± 0.04	(2)	a	0.53 ± 0.10	(2)	a
	**Ile**	0.26 ± 0.03	b	0.24 ± 0.03		b	0.96 ± 0.09	(4)	a	0.99 ± 0.17	(4)	a
	**Met**	0.056 ± 0.004	b	0.045 ± 0.006		b	0.11 ± 0.01	(2)	a	0.10 ± 0.01	(2)	a
	**Phe**	0.17 ± 0.02	b	0.19 ± 0.02		b	0.51 ± 0.05	(3)	a	0.36 ± 0.08	(2)	a
	**Trp**	0.32 ± 0.04	b	0.29 ± 0.05		b	0.89 ± 0.08	(3)	a	0.60 ± 0.13		ab
	**His**	0.64 ± 0.11	b	0.79 ± 0.21		b	1.99 ± 0.21	(3)	ab	3.11 ± 0.61	(5)	a
	**Tyr**	0.15 ± 0.03	ab	0.10 ± 0.02		b	0.27 ± 0.03	(2)	a	0.28 ± 0.07		a
	**Val**	0.67 ± 0.10	b	0.60 ± 0.06		b	2.20 ± 0.13	(3)	a	1.96 ± 0.34	(3)	a
	**Gln**	0.45 ± 0.08	a	0.57 ± 0.09		a	0.78 ± 0.26		a	0.49 ± 0.16		a
	**Pro**	0.47 ± 0.07	c	6.86 ± 1.20	(14)	b	13.56 ± 0.92	(29)	a	14.25 ± 1.21	(30)	a
	**Asn**	12.8 ± 3.3	b	14.2 ± 3.6		b	44.1 ± 2.7	(3)	a	24.9 ± 5.1		b
	**GABA**	1.16 ± 0.12	b	0.87 ± 0.09		b	1.18 ± 0.05		b	3.27 ± 0.57	(3)	a
	**Thr**	1.66 ± 0.28	ab	0.94 ± 0.05		b	2.31 ± 0.19		a	2.11 ± 0.36		a
	**Ser**	2.1 ± 0.2	bc	1.7 ± 0.1		c	3.5 ± 0.1	(2)	a	3.2 ± 0.4	(2)	ab
	**Gly**	0.25 ± 0.03	b	0.24 ± 0.03		b	0.50 ± 0.04	(2)	a	0.76 ± 0.12	(3)	a
	**Ala**	0.75 ± 0.13	bc	0.60 ± 0.08		c	1.19 ± 0.08	(2)	b	3.48 ± 0.38	(5)	a
	**Glu**	0.66 ± 0.18	b	0.97 ± 0.09		ab	1.37 ± 0.13	(2)	a	1.10 ± 0.18		ab
	**Asp**	0.43 ± 0.06	b	0.45 ± 0.04		b	1.00 ± 0.11	(2)	a	0.64 ± 0.07		b
	**TOTAL**	**23.5 ± 4.3**	**c**	**30.3 ± 5.2**		**bc**	**78.0 ± 3.2**	**(3)**	**a**	**63.1 ± 11.4**	**(3)**	**ab**
