# Supplementary material for: Strategies to Apply Water-Deficit Stress: Similarities and Disparities at the Whole Plant Metabolism Level in Medicago truncatula
Source: Int J Mol Sci. 2021 Mar 10;22(6):2813. doi: 10.3390/ijms22062813 (PMC8002188; doi:10.3390/ijms22062813)
Supplement: Supplementary file 1 [file ijms-22-02813-s001.zip › ijms-1103292-revision-suppl/Table S2.pdf]

Table S2. Effect of the different treatments on the individual and total inorganic ions in *M truncatula*. Absolute content of individual (as µmol g DW^{-1}) and total (as mmol g DW^{-1}) inorganic ions in leaves and roots under control and water-deficit stress conditions. Values represent the means ± SE (n = 5). Letters represent statistical differences (Tukey`s test, P <0.05) between treatments. Those inorganic ions showing more than a (+/-) two-fold change and significant differences between control and treatments (according to Student's t-test, P <0.05) are highlighted in red and blue, respectively. *No-W*, no-watering.

		Na^+	K^+	Cl^-	Mg^{2+}	Ca^{2+}	NH_4^+	NO_3^-	SO_4^-	PO_4^{3-}	TOTAL
**Leaf**	**Control**	19.1 ±3.3 b	516.5 ± 20.9 b	91.4 ± 6.1 b	186.0 ± 8.3 b	69.4 ± 8.6 ab	8.1 ± 0.9 a	30.7 ± 1.9 a	327.9 ± 13.9 a	39.0 ± 3.6 ab	1.29 ± 0.05 bc
	**NaCl**	376.8 ±66.3 a	578.2 ± 35.4 b	757.0 ± 58.7 a	217.9 ± 12.3 a	81.3 ± 3.0 a	13.7 ± 4.6 a	47.4 ± 8.8 a	206.7 ± 17.4 c	35.5 ± 3.3 ab	2.29 ± 0.15 a
	**No-W**	21.3 ±1.2 b	542.2 ± 16.3 b	84.6 ± 4.8 b	190.09 ± 6.6 b	80.9 ± 6.2 a	12.3 ± 2.8 a	50.6 ± 7.1 a	343.0 ± 20.0 a	53.0 ± 6.2 a	1.37 ± 0.02 b
	**PEG**	13.5 ±2.0 b	401.7 ± 18.8 c	77.3 ± 5.3 cb	133.7 ± 4.1 c	52.6 ± 7.0 b	11.2 ± 3.1 a	27.8 ± 3.1 a	266.1 ± 22.2 bc	32.4 ± 3.2 b	1.02 ± 0.04 c
**Root**	**Control**	78.0 ±13.1 b	332.1 ± 30.8 b	54.4 ± 7.8 b	54.7 ± 4.9 a	56.0 ± 10.9 a	9.3 ± 0.9 a	7.9 ± 1.7 b	273.0 ± 33.4 b	16.6 ± 3.1 ab	0.88 ± 0.06 b
	**NaCl**	776.7 ±42.6 a	183.5 ± 13.4 bc	744.8 ± 78.9 a	13.1 ± 0.6 b	12.1 ± 1.1 b	9.7 ± 1.1 a	6.2 ± 1.0 b	73.1 ± 7.9 c	9.7 ± 1.4 b	1.78 ± 0.12 a
	**No-W**	63.6 ±13.4 b	292.9 ± 56.4 bc	70.3 ± 5.2 b	50.1 ± 5.0 a	74.4 ± 15.7 a	7.5 ± 2.7 a	23.1 ± 3.7 a	413.9 ± 15.9 a	18.5 ± 0.6 a	1.02 ± 0.09 b
	**PEG**	13.9 ±2.1 b	121.9 ± 12.1 c	24.8 ± 1.9 c	13.1 ± 1.5 b	16.6 ± 3.8 b	8.2 ± 1.7 a	6.1 ± 0.7 b	50.3 ± 5.1 c	20.9 ± 1.6 a	0.28 ± 0.02 c
