# Supplementary material for: Strategies to Apply Water-Deficit Stress: Similarities and Disparities at the Whole Plant Metabolism Level in Medicago truncatula
Source: Int J Mol Sci. 2021 Mar 10;22(6):2813. doi: 10.3390/ijms22062813 (PMC8002188; doi:10.3390/ijms22062813)
Supplement: Supplementary file 1 [file ijms-22-02813-s001.zip › ijms-1103292-revision-suppl/Table S3.pdf]

Table S3. Effect of the different water stress treatments on the antioxidant composition of *M. truncatula*. Total ascorbate (Asc), glutathione (Glut) and homoglutathione (hGlut) contents (as mg antioxidant g DW^{-1}), as well as their reduced (ASC, GSH and hGSH, respectively), oxidized forms (DHA, GSSG and hGSSG, respectively) and reduced/total antioxidant content ratios in leaves (L) and roots (R) under control, NaCl, no watering (No-W) and PEG 6000 treatments. Values represent the means ± SE (n = 5). Letters represent statistical differences (Tukey's test, $p \leq 0.05$) between treatments. No reduced nor oxidized ascorbate data could be measured in the root tissue. *Asc*, ascorbate; *ASC*, reduced ascorbate; *DHA*, oxidized ascorbate; *Glut*, glutathione; *GSH*, reduced glutathione; *GSSG*, oxidized glutathione; *hGlut*, homoglutathione; *hGSH*, reduced homoglutathione; *hGSSG*, oxidized homoglutathione; *n.d.*, not detected.

		Control	NaCl	No-W	PEG
**Leaves**	**ASC**	2.7 ± 0.18 a	1.8 ± 0.08 bc	2.1 ± 0.09 b	1.5 ± 0.04 c
	**DHA**	0.71 ± 0.22 a	0.64 ± 0.08 a	0.94 ± 0.06 a	0.88 ± 0.08 a
	**Total Asc**	3.4 ± 0.09 a	2.4 ± 0.08 b	3.0 ± 0.11 a	2.4 ± 0.11 b
	**ASC/Total Asc**	0.79 ± 0.06 a	0.74 ± 0.03 a	0.69 ± 0.02 a	0.63 ± 0.02 a
	**GSH**	0.36 ± 0.05 a	0.24 ± 0.008 a	0.31 ± 0.05 a	0.23 ± 0.03 a
	**GSSG**	0.34 ± 0.09 a	0.30 ± 0.03 a	0.35 ± 0.07 a	0.52 ± 0.04 a
	**Total Glut**	0.70 ± 0.13 a	0.54 ± 0.03 a	0.67 ± 0.12 a	0.75 ± 0.07 a
	**GSH/Total Glut**	0.52 ± 0.03 a	0.44 ± 0.04 a	0.47 ± 0.01 a	0.30 ± 0.02 b
	**hGSH**	0.12 ± 0.00 b	0.20 ± 0.01 a	0.13 ± 0.02 b	0.11 ± 0.01 b
	**hGSSG**	0.16 ± 0.03 a	0.11 ± 0.04 a	0.14 ± 0.05 a	0.070 ± 0.015 a
	**Total hGlut**	0.29 ± 0.03 a	0.31 ± 0.04 a	0.27 ± 0.06 a	0.18 ± 0.002 a
	**hGSH/Total hGlut**	0.44 ± 0.04 a	0.66 ± 0.07 a	0.51 ± 0.06 a	0.62 ± 0.08 a
**Roots**	**ASC**	0.21 ± 0.03 a	0.14 ± 0.02 a	n.d.	n.d.
	**DHA**	0.37 ± 0.02 a	0.22 ± 0.02 b	n.d.	n.d.
	**Total Asc**	0.58 ± 0.01 a	0.36 ± 0.05 b	0.23 ± 0.02 c	0.25 ± 0.03 bc
	**ASC/Total Asc**	0.36 ± 0.05 a	0.39 ± 0.008 a	n.d.	n.d.
	**GSH**	0.10 ± 0.02 b	0.066 ± 0.011 b	0.19 ± 0.04 a	0.063 ± 0.018 b
	**GSSG**	0.073 ± 0.026 b	0.10 ± 0.01 b	0.29 ± 0.06 a	0.14 ± 0.06 ab
	**Total Glut**	0.19 ± 0.02 b	0.17 ± 0.02b	0.45 ± 0.04 a	0.21 ± 0.08 b
	**GSH/Total Glut**	0.65 ± 0.09 a	0.39 ± 0.04 a	0.36 ± 0.09 a	0.38 ± 0.09 a
	**hGSH**	0.14 ± 0.02 b	0.11 ± 0.01 b	0.37 ± 0.06 a	0.12 ± 0.02 b
	**hGSSG**	0.35 ± 0.10 b	0.16 ± 0.03 b	0.86 ± 0.18 a	0.20 ± 0.04 b
	**Total hGlut**	0.52 ± 0.11 b	0.28 ± 0.03 b	1.2 ± 0.23 a	0.32 ± 0.06 b
	**hGSH/Total hGlut**	0.36 ± 0.06 a	0.42 ± 0.06 a	0.32 ± 0.04 a	0.38 ± 0.07 a
