# Supplementary material for: Strategies to Apply Water-Deficit Stress: Similarities and Disparities at the Whole Plant Metabolism Level in Medicago truncatula
Source: Int J Mol Sci. 2021 Mar 10;22(6):2813. doi: 10.3390/ijms22062813 (PMC8002188; doi:10.3390/ijms22062813)
Supplement: Supplementary file 1 [file ijms-22-02813-s001.zip › ijms-1103292-revision-suppl/Table S4-2.pdf]

Table S4. Loading values resulting from the Principal Component Analyses (PCA) performed between treatments. Loading values in the first PC for all parameters after performing a PCA between all treatments, no-irrigated (No-W) and PEG 6000 treated plants and between NaCl and No-W plants, respectively. *aa*, amino acids; *AAT*, aspartate aminotransferase; *act*, activity; *ASC*, reduced ascorbate; *asc*, ascorbate; *DHA*, oxidized ascorbate; *ET*, evapotranspiration; *GDH*, glutamate dehydrogenase; *glut*; glutathione; *GOGAT*, glutamate synthase; *G6PDH*, glucose-6-phosphate dehydrogenase; g_s, leaf conductivity; *GS*, glutamine synthetase; *GSH*, reduced glutathione; *GSSG*, oxidized glutathione; *hglut*; homoglutathione; *hGSH*, reduced homoglutathione; *hGSSG*, oxidized homoglutathione; *IDH*, isocitrate dehydrogenase; *INV*, alkaline invertase; *α-KG*, α-ketoglutarate; *L*, leaves; *OAT*, ornithine aminotransferase; *P*, phloem sap; *P5CS*, Δ^1-1-pyrroline-5-carboxylate synthase; *ProDH*, proline dehydrogenase; *prot*, protein; *R*, roots; *SuSy*, sucrose synthase; *WC*, water content; Ψ_{leaf}, leaf water potential.

All treatments		No-W vs PEG		NaCl vs No-W	
**Parameter**	**Loading value**	**Parameter**	**Loading value**	**Parameter**	**Loading value**
Starch (L)	-0.81534803	Starch (L)	-0.8877786	g_s	-0.577511924
g_s	-0.39652764	Fructose (R)	-0.14369507	Fructose (R)	0.224002466
Na^+ (R)	-0.13963575	Glucose (R)	-0.12566793	Na^+ (L)	-0.212196576
Pro h	0.10133519	Succinate (R)	0.12546307	Glucose (R)	0.203600727
Cl^- (R)	-0.09635565	SO_4^{2-} (R)	-0.12221486	Na^+ (R)	-0.197693913
Pro (R)	0.09619082	Ca^{2+} (R)	-0.09168435	Cl^- (R)	-0.171581146
Succinate (R)	0.09372189	Na^+ (R)	-0.08548867	Cl^- (L)	-0.164077825
Na^+ (L)	-0.08651566	hGSSG (R)	-0.085019	Starch (L)	-0.138109254
Ala (R)	0.08131573	Mg^{2+} (R)	-0.07783645	SO_4^{2-} (R)	0.134215402
Citrate (L)	-0.07886668	Total hglut (R)	-0.07729113	Ca^{2+} (R)	0.128653142
Total ions (R)	-0.07334973	NO_3^- (R)	-0.07581081	hGSSG (R)	0.122022681
His (R)	0.07006663	Total ions (R)	-0.0754448	Total hglut (R)	0.111009156
Ile (R)	0.06372335	hGSH (R)	-0.07467011	Ile (R)	0.105989125
His (L)	0.05800341	GSH (R)	-0.07278017	Citrate (L)	-0.102625271
Cl^- (L)	-0.05765546	Pro (L)	0.07221624	NO_3^- (R)	0.101899434
Sucrose (L)	0.05757817	Sucrose (L)	0.0665744	Mg^{2+} (R)	0.100901666
Phe (L)	0.0572286	INV act	-0.06209162	Glucose (L)	0.100769135
GABA (R)	0.05620323	Ala (R)	0.06109065	Val (R)	0.099912917
Gly (R)	0.05329359	OAT act (L)	-0.06056675	Ser (P)	-0.099684695
Val (R)	0.05232892	Total glut (R)	-0.05991987	Asn (R)	0.097426343
OAT act (L)	-0.05176269	Thr (L)	-0.0596395	Gln (P)	-0.097122108
SO_4^{2-} (R)	-0.05044332	Cl^- (R)	-0.05929589	Ser (L)	-0.096310933
Trp (L)	0.04774066	GSSG (R)	-0.05646363	Malate (R)	0.095883904
Arg (L)	0.04681946	GABA (R)	0.05424258	hGSH (R)	0.091603431
Arg (R)	0.04487833	K^+ (R)	-0.04671397	Trp (R)	0.087038767
Pro (P)	0.04372798	Glucose (L)	-0.0466086	Pro (P)	-0.086296972
Val (L)	0.04274874	Asn (P)	0.04447439	Fructose (L)	0.085593884
Leu (R)	0.04182452	Gln (P)	0.04375904	Arg (L)	0.08270638
Asp (P)	0.04180983	Asn (R)	-0.0389899	GSSG (R)	0.078964064
Total aa (R)	0.04145664	Citrate (L)	-0.03861317	GSH (R)	0.07871468
ET	-0.03730729	GABA (P)	0.03736114	Gln (L)	0.078584985
G6PDH act (R)	-0.03642413	Asp (L)	-0.03712184	Total glut (R)	0.078382578
Sucrose (R)	0.03618091	hGSSG (L)	-0.03603466	α-KG (L)	-0.078033124
Tyr (R)	0.03617831	Starch (R)	0.03520256	His (R)	0.077902349

Mg^{2+} (R)	-0.03574153	Fructose (L)	-0.03509003	α-KG (R)	0.077863422
K^{+} (R)	-0.03464785	His (L)	0.03336244	Phe (R)	0.077444704
Asp (L)	-0.03448404	Gln (R)	-0.03320122	Leu (R)	0.07660609
Met (R)	0.03333049	Leu (L)	-0.03256183	Total aa (R)	0.075955441
Asn (R)	0.03327641	NO_3^- (L)	-0.03201617	Lys (R)	0.074631052
Lys (R)	0.03296304	Glu (L)	-0.03176135	Tyr (R)	0.072269889
SuSy act (R)	-0.03160218	Malate (R)	-0.03097384	GABA (P)	-0.069238338
hGSSG (L)	-0.03109651	G6PDH act (L)	-0.03053895	Arg (P)	-0.068861311
Ca^{2+} (R)	-0.03028826	Ile (L)	-0.02982574	Met (R)	0.068809806
Glucose (L)	0.02965629	Trp (R)	-0.02980461	Leu (L)	0.067735998
Phe (R)	0.02959594	Tyr (P)	0.02972483	Thr (R)	0.067191527
Trp (R)	0.02913745	Ser (L)	0.02970315	Asn (L)	0.066638938
Total asc (R)	-0.0290198	Total aa (P)	0.0280889	Val (L)	0.064630625
Ψ_{leaf}	0.02880529	Malate (L)	-0.02788048	G6PDH act (R)	-0.062601772
α-KG (L)	-0.02833398	Na^{+} (L)	-0.02746197	Starch (R)	-0.061863328
Gly (L)	0.02771831	Ser (P)	0.02718649	Asp (R)	0.061175567
Asn (P)	0.02678959	PO_4^{3-} (L)	-0.02699422	Ile (L)	0.059828832
PO_4^{3-} (R)	0.02652051	Asp (R)	-0.02675986	Met (L)	0.059460766
Leu (P)	-0.02651475	Pro (P)	0.02652271	Phe (L)	0.057425908
INV act (R)	-0.02634016	Ca^{2+} (L)	-0.02633805	Ser (R)	0.05698747
Ile (P)	-0.02504429	GSH/total glut (L)	-0.02603465	Gly (R)	0.056335485
GSSG (L)	0.02501464	Lys (R)	-0.02571698	Arg (R)	0.055483533
Total ions (L)	-0.02499008	Phe (R)	-0.02564423	Lys (L)	0.054768813
Thr (L)	-0.0249019	GSSG (L)	0.02423802	Sucrose (L)	-0.054705276
Ser (R)	0.02483775	Met (L)	-0.02306044	INV act (R)	0.054667874
Fructose (R)	-0.02442944	Citrate (R)	-0.02236334	Pro (R)	0.054459571
P5CS act (R)	0.02389789	Arg (P)	0.02173129	PO_4^{3-} (R)	0.053822558
ProDH (R)	0.02376065	Gly (P)	0.02115352	Ala (R)	0.053569033
Thr (R)	0.02309042	Gly (R)	0.02071017	Tyr (L)	0.052829715
GSH/total glut (L)	-0.0226499	Ile (P)	-0.02058836	Thr (L)	0.049815571
Total hglut (L)	-0.02256089	Mg^{2+} (L)	-0.02028353	Leu (P)	-0.046046653
α-KG (R)	0.0225533	Gln (L)	-0.02022112	Total ions (R)	-0.043092963
Lys (L)	0.02214824	Phe (L)	0.01948603	Tyr (P)	-0.042839216
Gln (L)	0.0221376	Total hglut (L)	-0.01923566	Trp (L)	0.041763246
Citrate (R)	-0.02158719	Lys (L)	-0.01844734	Total aa (P)	-0.040173072
GOGAT act (R)	-0.02155164	ASC (L)	-0.01842526	Ala (P)	-0.038868568
Total aa (L)	0.02146917	Asn (L)	-0.01840857	Total ions (L)	-0.03817593
GSSG (R)	0.02140409	K^{+} (L)	-0.01798545	GOGAT act (L)	-0.038015493
Fructose (L)	0.02138864	His (R)	0.01766514	SO_4^{2-} (L)	0.037037952
Malate (L)	-0.02068362	GSH (L)	-0.01749408	NH_4^{+} (R)	-0.036758314
Met (L)	0.0201942	Total aa (R)	-0.0174007	GABA (L)	0.036626469
Mg^{2+} (L)	-0.01967808	Total ions (L)	-0.01739975	His (L)	0.036394586
GSH (R)	-0.01955265	Succinate (L)	-0.0167867	Thr (P)	-0.035364138
Glu (L)	-0.01854577	Thr (P)	0.01649375	Total asc (R)	-0.034793924
Succinate (L)	-0.01853404	GOGAT act (L)	0.016336	Met (P)	-0.034654353
ASC (L)	-0.01838927	Glu (R)	-0.01631494	Sucrose (R)	0.034324412
Asp (R)	0.01824464	Ala (L)	-0.0146028	Gly (P)	-0.03306943
Ser (P)	-0.01822614	Root WC	0.01419282	hGSH (L)	-0.032445452
Ca^{2+} (L)	-0.01812861	Total asc (L)	-0.01353035	ProDH act (R)	0.032259808

Lys (P)	-0.01732111	Trp (L)	0.01331278	Gly (L)	0.032238828
Starch (R)	-0.01700272	Arg (L)	0.01322875	Pro (L)	-0.031577653
Glu (R)	0.01627183	Lys (P)	-0.01305654	Val (P)	0.031203951
Tyr (L)	0.01531889	SO_4^{2-} (L)	-0.01288158	DHA (L)	0.03020021
Total aa (P)	0.01531399	Ala (P)	0.01285062	Asn (P)	-0.030076143
hGSH (L)	-0.01508114	His (P)	0.01276221	G6PDH act (L)	0.029823204
GABA (L)	0.01507409	Root biomass	-0.0121317	Asp (P)	0.029571442
K^+ (L)	-0.01485324	AAT act (R)	0.01202279	K^+ (R)	0.028585557
DHA (L)	0.01410628	Val (R)	-0.01186893	GOGAT act (R)	-0.027519782
Gln (P)	0.0140909	Tyr (L)	-0.01165093	PO_4^{3-} (L)	0.026944284
GABA (P)	0.01399367	α-KG (R)	-0.01106011	Glu (R)	0.026895378
GSH/total glut (R)	-0.0139049	hGSH/total hglut (L)	0.01084305	GDH act (R)	-0.025792535
Ile (L)	0.01342223	NH_4^+ (R)	0.01060226	Trp (P)	0.025738405
AAT act (R)	-0.01295125	Gly (L)	0.01036693	SuSy act (R)	-0.025636227
hGSH (R)	-0.01268598	NH_4^+ (L)	-0.0103402	ET	-0.025548568
Malate (R)	0.01206245	Asp (P)	0.01029509	GABA (R)	0.02553827
GSH (L)	-0.01174776	ProDH act (L)	0.01015221	P5CS act (R)	0.021054268
Asn (L)	0.01172686	Thr (R)	-0.01004851	GSH (L)	0.020570661
NO_3^- (L)	-0.01156894	Root/shoot ratio	-0.00990652	OAT act (L)	-0.020360233
NH_4^+ (R)	-0.01103665	AAT act (L)	0.00941221	hGSH/total hglut (L)	-0.020025286
Total glut (L)	0.01045201	SuSy act (R)	0.00923385	hGSH/total hglut (R)	-0.019610217
OAT act (R)	0.01036028	GOGAT act (R)	-0.00905751	AAT act (R)	-0.019594212
Soluble prot (R)	-0.01024324	Ser (R)	-0.00900478	P5CS act (L)	-0.019475326
GOGAT act (L)	-0.00985651	IDH act (R)	0.00878415	Total aa (L)	0.018975065
Gln (R)	-0.00952258	Total aa (L)	0.00824142	Sucrose (P)	0.018900872
Glu (P)	0.00939953	G6PDH act (R)	-0.00818711	hGSSG (L)	0.018638512
AAT act (L)	0.00938694	Total glut (L)	0.0081641	Ala (L)	0.017493033
Total asc (L)	-0.00928642	Leaf WC	-0.007071	NO_3^- (L)	0.017337924
Ser (L)	-0.00924999	PO_4^{3-} (R)	0.00704527	Root/shoot ratio	0.016284384
ASC/total asc (L)	-0.00908832	Met (R)	-0.00697356	Root WC	-0.016247138
G6PDH act (L)	-0.00829122	Total biomass	-0.00695937	Total asc (L)	0.016215034
Met (P)	-0.00819351	hGSH (L)	-0.00695254	Total glut (L)	0.015836376
Glucose (R)	-0.00817222	GDH act (R)	0.00682392	Glu (L)	0.015806757
Leaf WC	-0.00790642	Leu (P)	0.00676422	Gln (R)	0.01442023
Arg (P)	-0.00785961	Phe (P)	0.00664416	Root biomass	0.013827458
hGSSG (R)	-0.0073966	P5CS act (R)	0.00662805	IDH act (L)	-0.013615206
PO_4^{3-} (L)	-0.00735788	OAT act (R)	0.00651063	Phe (P)	-0.013379837
Gly (P)	0.00705811	GSH/total glut (R)	0.0063357	ProDH act (L)	0.013081465
hGSH/total hglut (L)	0.00693307	GS act (L)	-0.00593337	GSSG (L)	0.012187883
Total hglut (R)	-0.00670608	Tyr (R)	-0.00584023	Ile (P)	0.012096376
Leu (L)	0.00662279	Leu (R)	-0.00556016	OAT act (R)	-0.01198919
ProDH act (L)	0.00651214	Arg (R)	-0.00547648	IDH act (R)	-0.011690066
hGSH/total hglut (R)	-0.0061584	Val (P)	-0.00528931	Total hglut (L)	-0.011561997
Phe (P)	-0.00575924	Met (P)	0.00519184	ASC (L)	0.010791638
Shoot biomass	-0.00557483	Soluble prot (L)	0.00508262	Asp (L)	0.010412812
P5CS act (L)	-0.00518319	Cl^- (L)	-0.00497952	Mg^{2+} (L)	-0.010178277
His (P)	-0.00457464	ASC/total asc (L)	-0.00484322	GS act (L)	0.009864848

Total biomass	-0.00439455	Total asc (R)	0.00464961	Succinate (L)	-0.00955885
Chlorophyll	-0.00402871	DHA (L)	-0.00402801	GSH/total glut (R)	-0.00939106
Sucrose (P)	0.00390169	Soluble prot (R)	-0.00396818	AAT act (L)	0.007680379
Root/shoot ratio	0.00363505	α-KG (L)	0.00374766	Malate (L)	-0.007493607
GDH act (R)	-0.00326866	hGSH/total hglut (R)	0.00351712	Citrate (R)	0.007438883
IDH act (L)	-0.00311892	Chlorophyll	-0.00350375	Soluble prot (R)	-0.007273566
Total glut (R)	-0.00299956	Pro (R)	-0.00340538	Succinate (R)	-0.00717161
Root biomass	-0.0025657	IDH act (L)	-0.00312762	Total biomass	0.005484732
NO_3^- (R)	-0.00247509	Ile (R)	-0.00287927	ASC/total asc (L)	-0.00545219
Trp (P)	-0.00228543	GDH act (L)	0.00255387	GSH/total glut (L)	0.004672201
Soluble prot (L)	0.00205727	Shoot biomass	-0.00222518	K^+ (L)	-0.003952141
Tyr (P)	0.00197497	Sucrose (P)	-0.00215362	Glu (P)	-0.003815143
Ala (P)	0.0018538	P5CS act (L)	-0.00171412	Shoot biomass	-0.00373056
GS act (L)	0.00152989	Trp (P)	0.00146537	NH_4^+ (L)	-0.002879225
SO_4^{2-} (L)	0.001466	Glu (P)	-0.00113892	Leaf WC	-0.002258014
Root WC	0.00135001	Sucrose (R)	0.00108485	Chlorophyll	-0.001446955
GDH act (L)	0.00112902	Val (L)	0.00105831	Ca^{2+} (L)	-0.00142577
Val (P)	0.00076596	GABA (L)	0.00070838	Soluble prot (L)	0.001302616
Ala (L)	-0.00055575	ET	0.00025655	Lys (P)	0.001208123
Thr (P)	0.00046669	ProDH act (R)	-0.00017074	Ψ_{leaf}	0.000263171
IDH act (R)	0.00045863	Ψ_{leaf}	-7.78E-05	His (P)	-0.000120468
NH_4^+ (L)	-0.00040771	g_s	0	GDH act (L)	-3.13E-05
