# Supplementary material for: Strategies to Apply Water-Deficit Stress: Similarities and Disparities at the Whole Plant Metabolism Level in Medicago truncatula
Source: Int J Mol Sci. 2021 Mar 10;22(6):2813. doi: 10.3390/ijms22062813 (PMC8002188; doi:10.3390/ijms22062813)
Supplement: Supplementary file 1 [file ijms-22-02813-s001.zip › ijms-1103292-revision-suppl/Table S5.pdf]

Organ	Type of measurement	Parameter	Units	Control	Control
**Plant**	**Physiological measurements**	**g_s**	($cm\ s^{-1}$)	0.07	0.08
**Plant**	**Physiological measurements**	**ET**	($g\ H_2O\ day^{-1}\ plant^{-1}$)		182.70
**Leaf**	**Physiological measurements**	**Chlorophyll**	(SPAD units)	65.20	
**Plant**	**Physiological measurements**	**Ψ_{leaf}**	(MPa)	-0.49	-0.51
**Leaf**	**Physiological measurements**	**Water content**	(%)	80.01	81.17
**Root**	**Physiological measurements**	**Water content**	(%)	83.79	
**Plant**	**Physiological measurements**	**Total biomass**	(g DW)	18.26	14.37
**Leaf**	**Physiological measurements**	**Shoot biomass**	(g DW)	10.11	8.32
**Root**	**Physiological measurements**	**Root biomass**	(g DW)	8.15	6.05
**Plant**	**Physiological measurements**	**Root/shoot ratio**		0.81	0.73
**Leaf**	**Carbohydrates**	**Fructose**	($\mu mol\ g\ DW^{-1}$)	5.75	2.43
**Leaf**	**Carbohydrates**	**Glucose**	($\mu mol\ g\ DW^{-1}$)	3.99	2.31
**Leaf**	**Carbohydrates**	**Sucrose**	($\mu mol\ g\ DW^{-1}$)	15.78	13.96
**Leaf**	**Carbohydrates**	**Starch**	($\mu mol\ g\ DW^{-1}$)	157.92	82.40
**Leaf**	**Carbohydrates**	**Succinate**	($\mu mol\ g\ DW^{-1}$)	0.10	0.10
**Leaf**	**Carbohydrates**	**Malate**	($\mu mol\ g\ DW^{-1}$)	5.83	3.64
**Leaf**	**Carbohydrates**	**α-KG**	($\mu mol\ g\ DW^{-1}$)	0.04	0.04
**Leaf**	**Carbohydrates**	**Citrate**	($\mu mol\ g\ DW^{-1}$)	2.12	1.72
**Phloem sap**	**Carbohydrates**	**Sucrose**	($\mu mol\ g\ DW^{-1}$)	15.62	21.03
**Root**	**Carbohydrates**	**Fructose**	($\mu mol\ g\ DW^{-1}$)	37.57	41.39
**Root**	**Carbohydrates**	**Glucose**	($\mu mol\ g\ DW^{-1}$)	16.02	26.39
**Root**	**Carbohydrates**	**Sucrose**	($\mu mol\ g\ DW^{-1}$)	16.08	20.24
**Root**	**Carbohydrates**	**Starch**	($\mu mol\ g\ DW^{-1}$)	16.43	10.67
**Root**	**Carbohydrates**	**Succinate**	($\mu mol\ g\ DW^{-1}$)	0.12	0.11
**Root**	**Carbohydrates**	**Malate**	($\mu mol\ g\ DW^{-1}$)	0.85	1.05
**Root**	**Carbohydrates**	**α-KG**	($\mu mol\ g\ DW^{-1}$)	0.03	0.04
**Root**	**Carbohydrates**	**Citrate**	($\mu mol\ g\ DW^{-1}$)	0.66	0.58
**Leaf**	**Soluble protein**	**Total**	($mg\ g\ DW^{-1}$)	149.07	166.90
**Root**	**Soluble protein**	**Total**	($mg\ g\ DW^{-1}$)	25.60	28.70
**Leaf**	**Soluble aa**	**Arg**	($\mu mol\ g\ DW^{-1}$)	0.29	0.59
**Leaf**	**Soluble aa**	**Lys**	($\mu mol\ g\ DW^{-1}$)	0.37	0.31
**Leaf**	**Soluble aa**	**Leu**	($\mu mol\ g\ DW^{-1}$)	0.63	0.67
**Leaf**	**Soluble aa**	**Ile**	($\mu mol\ g\ DW^{-1}$)	0.51	0.55
**Leaf**	**Soluble aa**	**Met**	($\mu mol\ g\ DW^{-1}$)	0.13	0.10
**Leaf**	**Soluble aa**	**Phe**	($\mu mol\ g\ DW^{-1}$)	0.60	0.64
**Leaf**	**Soluble aa**	**Trp**	($\mu mol\ g\ DW^{-1}$)	0.68	0.31
**Leaf**	**Soluble aa**	**His**	($\mu mol\ g\ DW^{-1}$)	0.54	0.64
**Leaf**	**Soluble aa**	**Tyr**	($\mu mol\ g\ DW^{-1}$)	0.33	0.44
**Leaf**	**Soluble aa**	**Val**	($\mu mol\ g\ DW^{-1}$)	1.43	1.49
**Leaf**	**Soluble aa**	**Gln**	($\mu mol\ g\ DW^{-1}$)	0.73	0.75
**Leaf**	**Soluble aa**	**Pro**	($\mu mol\ g\ DW^{-1}$)	1.97	1.87
**Leaf**	**Soluble aa**	**Asn**	($\mu mol\ g\ DW^{-1}$)	49.66	18.08
**Leaf**	**Soluble aa**	**GABA**	($\mu mol\ g\ DW^{-1}$)	0.52	0.56
**Leaf**	**Soluble aa**	**Thr**	($\mu mol\ g\ DW^{-1}$)	2.26	2.38
**Leaf**	**Soluble aa**	**Ser**	($\mu mol\ g\ DW^{-1}$)	1.83	4.98
**Leaf**	**Soluble aa**	**Gly**	($\mu mol\ g\ DW^{-1}$)	0.29	0.18
**Leaf**	**Soluble aa**	**Ala**	($\mu mol\ g\ DW^{-1}$)	2.39	1.18
**Leaf**	**Soluble aa**	**Glu**	($\mu mol\ g\ DW^{-1}$)	11.28	9.27
**Leaf**	**Soluble aa**	**Asp**	($\mu mol\ g\ DW^{-1}$)	4.67	4.11
**Leaf**	**Soluble aa**	**Total**	($\mu mol\ g\ DW^{-1}$)	81.11	49.09
**Phloem sap**	**Soluble aa**	**Arg**	($\mu mol\ g\ DW^{-1}$)	0.05	0.07
**Phloem sap**	**Soluble aa**	**Lys**	($\mu mol\ g\ DW^{-1}$)	0.24	0.39

Organ	Type of measurement	Abreviation	Units	Control	Control
Phloem sap	Soluble aa	Leu	(µmol g DW^{-1})	0.15	0.25
Phloem sap	Soluble aa	Ile	(µmol g DW^{-1})	0.19	0.38
Phloem sap	Soluble aa	Met	(µmol g DW^{-1})	0.02	0.02
Phloem sap	Soluble aa	Phe	(µmol g DW^{-1})	0.08	0.19
Phloem sap	Soluble aa	Trp	(µmol g DW^{-1})	0.01	0.04
Phloem sap	Soluble aa	His	(µmol g DW^{-1})	0.08	0.17
Phloem sap	Soluble aa	Tyr	(µmol g DW^{-1})	0.09	0.11
Phloem sap	Soluble aa	Val	(µmol g DW^{-1})	0.50	0.92
Phloem sap	Soluble aa	Gln	(µmol g DW^{-1})	0.24	0.15
Phloem sap	Soluble aa	Pro	(µmol g DW^{-1})	0.18	0.25
Phloem sap	Soluble aa	Asn	(µmol g DW^{-1})	3.47	5.48
Phloem sap	Soluble aa	GABA	(µmol g DW^{-1})	0.61	0.71
Phloem sap	Soluble aa	Thr	(µmol g DW^{-1})	0.16	0.37
Phloem sap	Soluble aa	Ser	(µmol g DW^{-1})	0.44	1.14
Phloem sap	Soluble aa	Gly	(µmol g DW^{-1})	0.06	0.07
Phloem sap	Soluble aa	Ala	(µmol g DW^{-1})	0.21	0.29
Phloem sap	Soluble aa	Glu	(µmol g DW^{-1})	0.25	0.48
Phloem sap	Soluble aa	Asp	(µmol g DW^{-1})	0.28	0.68
Phloem sap	Soluble aa	Total	(µmol g DW^{-1})	7.34	12.16
Root	Soluble aa	Arg	(µmol g DW^{-1})	0.08	0.21
Root	Soluble aa	Lys	(µmol g DW^{-1})	0.15	0.39
Root	Soluble aa	Leu	(µmol g DW^{-1})	0.16	0.30
Root	Soluble aa	Ile	(µmol g DW^{-1})	0.18	0.32
Root	Soluble aa	Met	(µmol g DW^{-1})	0.04	0.06
Root	Soluble aa	Phe	(µmol g DW^{-1})	0.12	0.23
Root	Soluble aa	Trp	(µmol g DW^{-1})	0.23	0.42
Root	Soluble aa	His	(µmol g DW^{-1})	0.51	0.95
Root	Soluble aa	Tyr	(µmol g DW^{-1})	0.09	0.27
Root	Soluble aa	Val	(µmol g DW^{-1})	0.43	0.88
Root	Soluble aa	Gln	(µmol g DW^{-1})	0.33	0.65
Root	Soluble aa	Pro	(µmol g DW^{-1})	0.32	0.52
Root	Soluble aa	Asn	(µmol g DW^{-1})	4.41	16.11
Root	Soluble aa	GABA	(µmol g DW^{-1})	0.80	1.28
Root	Soluble aa	Thr	(µmol g DW^{-1})	0.95	1.52
Root	Soluble aa	Ser	(µmol g DW^{-1})	1.63	2.23
Root	Soluble aa	Gly	(µmol g DW^{-1})	0.23	0.28
Root	Soluble aa	Ala	(µmol g DW^{-1})	0.37	0.72
Root	Soluble aa	Glu	(µmol g DW^{-1})	0.43	1.16
Root	Soluble aa	Asp	(µmol g DW^{-1})	0.30	0.46
Root	Soluble aa	Total	(µmol g DW^{-1})	11.76	28.95
Leaf	Ions	Na^+	(nmol g DW^{-1})	0.02	0.03
Leaf	Ions	NH_4^+	(nmol g DW^{-1})	0.01	0.01
Leaf	Ions	K^+	(nmol g DW^{-1})	0.46	0.58
Leaf	Ions	Mg^{2+}	(nmol g DW^{-1})	0.17	0.21
Leaf	Ions	Ca^{2+}	(nmol g DW^{-1})	0.07	0.10
Leaf	Ions	Cl^-	(nmol g DW^{-1})	0.06	0.09
Leaf	Ions	NO_3^-	(nmol g DW^{-1})	0.03	0.03
Leaf	Ions	SO_4^{2-}	(nmol g DW^{-1})	0.31	0.37
Leaf	Ions	PO_4^{3-}	(nmol g DW^{-1})	0.04	0.04
Leaf	Ions	Total	(µmol g DW^{-1})	1.21	1.45
Root	Ions	Na^+	(nmol g DW^{-1})	0.09	0.12

Organ	Type of measurement	Abreviation	Units	Control	Control
**Root**	**Ions**	**NH_4^+**	(nmol g DW^{-1})	0.01	0.01
**Root**	**Ions**	**K^+**	(nmol g DW^{-1})	0.33	0.44
**Root**	**Ions**	**Mg^{2+}**	(nmol g DW^{-1})	0.06	0.06
**Root**	**Ions**	**Ca^{2+}**	(nmol g DW^{-1})	0.06	0.06
**Root**	**Ions**	**Cl^-**	(nmol g DW^{-1})	0.04	0.09
**Root**	**Ions**	**NO_3^-**	(nmol g DW^{-1})	0.01	0.01
**Root**	**Ions**	**SO_4^{2-}**	(nmol g DW^{-1})	0.32	0.42
**Root**	**Ions**	**PO_4^{3-}**	(nmol g DW^{-1})	0.02	0.01
**Root**	**Ions**	**Total**	(µmol g DW^{-1})	0.99	1.00
**Leaf**	**Antioxidants**	**ASC**	(mg g DW^{-1})	2.53	2.52
**Leaf**	**Antioxidants**	**DHA**	(mg g DW^{-1})	0.75	1.07
**Leaf**	**Antioxidants**	**Total Asc**	(mg g DW^{-1})	3.28	3.58
**Leaf**	**Antioxidants**	**ASC/Total Asc**		0.77	0.70
**Leaf**	**Antioxidants**	**GSH**	(mg g DW^{-1})	0.36	0.45
**Leaf**	**Antioxidants**	**GSSG**	(mg g DW^{-1})	0.28	0.52
**Leaf**	**Antioxidants**	**Total Glut**	(mg g DW^{-1})	0.64	0.96
**Leaf**	**Antioxidants**	**GSH/Total Glut**		0.57	0.46
**Leaf**	**Antioxidants**	**hGSH**	(mg g DW^{-1})	0.12	0.13
**Leaf**	**Antioxidants**	**hGSSG**	(mg g DW^{-1})	0.20	0.19
**Leaf**	**Antioxidants**	**Total hGlut**	(mg g DW^{-1})	0.32	0.32
**Leaf**	**Antioxidants**	**hGSH/Total hGlut**		0.38	0.40
**Root**	**Antioxidants**	**ASC**	(mg g DW^{-1})	0.27	
**Root**	**Antioxidants**	**DHA**	(mg g DW^{-1})	0.33	
**Root**	**Antioxidants**	**Total Asc**	(mg g DW^{-1})	0.60	
**Root**	**Antioxidants**	**ASC/Total Asc**		0.45	
**Root**	**Antioxidants**	**GSH**	(mg g DW^{-1})	0.12	
**Root**	**Antioxidants**	**GSSG**	(mg g DW^{-1})	0.04	
**Root**	**Antioxidants**	**Total Glut**	(mg g DW^{-1})	0.15	
**Root**	**Antioxidants**	**GSH/Total Glut**		0.76	
**Root**	**Antioxidants**	**hGSH**	(mg g DW^{-1})	0.16	
**Root**	**Antioxidants**	**hGSSG**	(mg g DW^{-1})	0.12	
**Root**	**Antioxidants**	**Total hGlut**	(mg g DW^{-1})	0.28	
**Root**	**Antioxidants**	**hGSH/Total hGlut**		0.56	
**Leaf**	**Enzymatic activities**	**G6PDH**	(nmol NADH min^{-1}	0.10	0.12
**Leaf**	**Enzymatic activities**	**IDH**	(nmol NADH min^{-1}	1.71	1.75
**Leaf**	**Enzymatic activities**	**AAT**	(nmol NADH min^{-1}	4.29	3.41
**Leaf**	**Enzymatic activities**	**GDH**	(nmol NADH min^{-1}	0.80	0.88
**Leaf**	**Enzymatic activities**	**GOGAT**	(nmol NADH min^{-1}	0.06	0.07
**Leaf**	**Enzymatic activities**	**GS**	(nmol NADH min^{-1}	0.03	0.03
**Leaf**	**Enzymatic activities**	**OAT**	(nmol NADH min^{-1}	0.34	0.27
**Leaf**	**Enzymatic activities**	**P5CS**	(nmol NADH min^{-1}	0.38	0.48
**Leaf**	**Enzymatic activities**	**ProDH**	(nmol NADH min^{-1}	0.27	0.31
**Root**	**Enzymatic activities**	**AlkINV**	(nmol NADH min^{-1}	3.24	3.32
**Root**	**Enzymatic activities**	**G6PDH**	(nmol NADH min^{-1}	4.23	3.79
**Root**	**Enzymatic activities**	**IDH**	(nmol NADH min^{-1}	6.05	5.99
**Root**	**Enzymatic activities**	**SuSy**	(nmol NADH min^{-1}	2.66	2.60
**Root**	**Enzymatic activities**	**AAT**	(nmol NADH min^{-1}	14.38	15.74
**Root**	**Enzymatic activities**	**GDH**	(nmol NADH min^{-1}	16.29	10.54
**Root**	**Enzymatic activities**	**GOGAT**	(nmol NADH min^{-1}	0.28	
**Root**	**Enzymatic activities**	**OAT**	(nmol NADH min^{-1}	1.63	1.40
**Root**	**Enzymatic activities**	**P5CS**	(nmol NADH min^{-1}	0.53	0.72

Organ	Type of measurement	Abreviation	Units	Control	Control
**Root**	**Enzymatic activities**	**ProDH**	(nmol NADH min^{-1} µ	0.49	0.57

Parameter	Control	Control	Control	Control	Control	Control	Control	Control	NaCl	NaCl
g_s	0.10	0.10	0.08						0.02	0.02
ET	158.20	168.50		181.74				179.40	59.25	56.45
Chlorophyll	62.20		64.50		64.90		63.40		66.00	
Ψ_{leaf}	-0.50	-0.50	-0.50						-1.70	-1.60
Water content	81.68	81.52	82.37	80.89	84.75	82.56	81.00	80.67	76.54	
Water content	83.13	84.95	80.63	82.38		80.00			88.82	
Total biomass	15.77	17.81		17.24					19.93	16.55
Shoot biomass	8.32	10.08	7.04	9.24	8.56	8.95	8.71		11.22	8.68
Root biomass	7.46	7.74		8.00					8.71	7.87
Root/shoot ratio	0.90	0.77		0.87					0.78	0.91
Fructose	6.01	4.00	12.54						2.32	7.69
Glucose	8.01	2.83	4.28						2.08	14.86
Sucrose	5.43	10.36	11.99						46.89	39.85
Starch	99.70	248.99	147.25						360.29	291.98
Succinate	0.19	0.10	0.10						0.15	0.09
Malate	9.67	2.38	1.58						6.32	3.51
α-KG	0.05	0.04	0.04						0.09	0.07
Citrate	5.86	1.18	0.61						4.34	2.12
Sucrose	15.18	24.21	13.32						26.57	16.45
Fructose	18.11	15.36	21.75						3.88	6.76
Glucose	13.19	12.73	13.06						5.99	4.83
Sucrose	10.96	19.66	13.45						22.60	31.94
Starch	10.15	8.82	5.77						6.22	8.93
Succinate	0.10	0.07	0.08						0.06	0.06
Malate	2.86	0.58	0.73						0.58	0.41
α-KG	0.02	0.02	0.03						0.01	0.01
Citrate	0.82	0.47	0.42						0.82	0.46
Total	128.86	160.03	146.87						143.51	147.54
Total	29.00								26.10	22.00
Arg	1.17	0.37	0.96						0.16	0.38
Lys	0.82	0.55	0.68						0.47	0.78
Leu	1.11	0.84	0.92						0.36	0.90
Ile	1.25	0.62	1.03						0.52	1.13
Met	0.15	0.14	0.12						0.09	0.20
Phe	1.08	0.71	0.96						0.51	1.77
Trp	0.50	0.53	0.48						0.49	1.31
His	0.65	0.55	0.61						0.28	1.26
Tyr	0.69	0.48	0.63						0.33	0.47
Val	3.44	1.59	3.02						1.50	2.87
Gln	0.87	0.91	0.78						0.49	0.78
Pro	2.24	1.88	1.48						22.23	26.80
Asn	51.28	35.36	87.21						15.39	68.47
GABA	1.18	0.51	0.50						0.35	0.80
Thr	3.31	2.46	2.39						1.42	2.09
Ser	8.96	4.07	3.30						2.59	21.80
Gly	0.37	0.34	0.28						0.16	0.33
Ala	3.46	1.83	1.63						2.10	1.65
Glu	16.79	10.90	8.47						8.73	9.26
Asp	7.89	4.75	4.04						3.12	2.21
Total	107.20	69.39	119.48						61.29	145.24
Arg	0.04	0.07	0.04						0.15	0.09
Lys	0.21	0.30	0.35						0.25	0.18

Abreviation	Control	Control	Control	Control	Control	Control	Control	Control	NaCl	NaCl
**Leu**	0.10	0.20	0.13						0.17	0.11
**Ile**	0.14	0.26	0.18						0.16	0.10
**Met**	0.01	0.02	0.01						0.02	0.01
**Phe**	0.06	0.10	0.08						0.09	0.07
**Trp**	0.01	0.03	0.02						0.02	0.01
**His**	0.08	0.11	0.06						0.06	0.08
**Tyr**	0.06	0.09	0.07						0.12	0.07
**Val**	0.64	0.73	0.50						0.49	0.29
**Gln**	0.04	0.15	0.14						0.39	0.68
**Pro**	0.09	0.13	0.18						6.03	3.11
**Asn**	1.98	5.19	3.02						4.05	2.37
**GABA**	0.69	0.56	0.39						2.74	1.61
**Thr**	0.21	0.28	0.19						0.43	0.23
**Ser**	0.38	0.53	0.28						1.48	0.89
**Gly**	0.04	0.04	0.03						0.11	0.06
**Ala**	0.23	0.30	0.20						0.69	0.44
**Glu**	0.46	0.56	0.36						1.24	0.56
**Asp**	1.28	1.20	0.51						2.80	0.92
**Total**	6.76	10.85	6.75						21.48	11.89
**Arg**	0.09	0.16	0.10						0.13	0.15
**Lys**	0.15	0.26	0.16						0.20	0.23
**Leu**	0.24	0.27	0.15						0.22	0.21
**Ile**	0.29	0.32	0.18						0.32	0.25
**Met**	0.05	0.07	0.05						0.05	0.03
**Phe**	0.16	0.22	0.14						0.21	0.17
**Trp**	0.24	0.39	0.31						0.23	0.19
**His**	0.33	0.79	0.62						0.63	0.43
**Tyr**	0.12	0.17	0.09						0.09	0.08
**Val**	0.79	0.80	0.44						0.73	0.62
**Gln**	0.24	0.58	0.43						0.33	0.46
**Pro**	0.40	0.70	0.43						4.90	5.55
**Asn**	23.07	13.66	6.76						7.33	14.60
**GABA**	1.54	1.17	1.02						0.71	0.94
**Thr**	1.76	2.64	1.44						1.10	0.96
**Ser**	2.36	2.67	1.41						1.60	1.57
**Gly**	0.30	0.30	0.15						0.22	0.19
**Ala**	1.01	1.05	0.57						0.40	0.83
**Glu**	0.21	0.99	0.52						0.66	0.99
**Asp**	0.57	0.55	0.26						0.31	0.51
**Total**	33.91	27.78	15.23						20.37	28.96
**Na^+**	0.02	0.02	0.01						0.15	0.32
**NH_4^+**	0.01	0.01	0.01						0.01	0.01
**K^+**	0.53	0.54	0.49						0.47	0.63
**Mg^{2+}**	0.17	0.20	0.18						0.19	0.21
**Ca^{2+}**	0.05	0.06	0.07						0.08	0.08
**Cl^-**	0.11	0.09	0.07	0.11	0.12	0.08	0.08	0.09		0.56
**NO_3^-**		0.03	0.03	0.03		0.03	0.02	0.04	0.02	0.02
**SO_4^{2-}**		0.34	0.25	0.31		0.33	0.34	0.37	0.13	0.25
**PO_4^{3-}**	0.05	0.04	0.02	0.03	0.04	0.03	0.04	0.06	0.03	0.04
**Total**	1.19	1.31	1.29						1.70	2.44
**Na^+**	0.04	0.07	0.07						0.79	0.72

Abreviation	Control	Control	Control	Control	Control	Control	Control	Control	NaCl	NaCl
**NH_4^+**	0.01	0.01	0.01						0.01	0.01
**K^+**	0.27	0.33	0.29						0.20	0.16
**Mg^{2+}**	0.05	0.07	0.04						0.02	0.01
**Ca^{2+}**	0.02	0.09	0.05						0.02	0.01
**Cl^-**	0.05	0.03	0.04	0.08	0.06	0.07	0.03		0.89	0.74
**NO_3^-**		0.00	0.00	0.02		0.00	0.01	0.01	0.01	0.01
**SO_4^{2-}**		0.26	0.27	0.32	0.13	0.40	0.16	0.20		0.07
**PO_4^{3-}**	0.01	0.01	0.02	0.03		0.02	0.03	0.01	0.01	0.02
**Total**	0.78	0.92	0.70						1.94	1.30
**ASC**	3.06								1.94	1.68
**DHA**	0.32								0.62	0.78
**Total Asc**	3.38								2.56	2.46
**ASC/Total Asc**	0.91								0.76	0.68
**GSH**	0.28								0.25	0.22
**GSSG**	0.24								0.25	0.37
**Total Glut**	0.51								0.49	0.59
**GSH/Total Glut**	0.54								0.50	0.38
**hGSH**	0.12								0.22	0.19
**hGSSG**	0.11								0.11	0.17
**Total hGlut**	0.22								0.33	0.37
**hGSH/Total hGlu**	0.53								0.66	0.53
**ASC**		0.15		0.21					0.12	0.18
**DHA**		0.41		0.37					0.19	0.27
**Total Asc**		0.56		0.58					0.30	0.45
**ASC/Total Asc**		0.27			0.36				0.38	0.41
**GSH**				0.09	0.15	0.02	0.13	0.08	0.04	0.08
**GSSG**				0.02	0.06		0.08	0.17	0.05	0.14
**Total Glut**				0.11	0.21		0.22	0.25	0.08	0.21
**GSH/Total Glut**				0.80	0.73		0.62	0.33	0.45	0.36
**hGSH**				0.15	0.16	0.06	0.19	0.15	0.06	0.14
**hGSSG**				0.17	0.37		0.71	0.39	0.14	0.06
**Total hGlut**				0.32	0.53		0.90	0.54	0.19	0.20
**hGSH/Total hGlut**				0.46	0.30		0.21	0.28	0.29	0.70
**G6PDH**	0.11	0.17							0.09	0.10
**IDH**	1.88	1.70	1.30						2.17	2.21
**AAT**	3.40	2.53	3.07						4.46	3.14
**GDH**	0.63	0.80	1.00						1.18	0.96
**GOGAT**	0.06								0.06	0.10
**GS**	0.03								0.03	0.02
**OAT**	0.39								0.45	0.46
**P5CS**	0.39								0.49	0.63
**ProDH**	0.19								0.18	0.19
**AlkINV**	2.49	2.96	2.70	2.57	1.51				1.31	1.37
**G6PDH**	4.05	4.41	4.36						4.30	5.96
**IDH**	6.03	10.32	8.77		8.35				6.00	7.44
**SuSy**	2.30	2.90	3.23						2.17	2.05
**AAT**	15.02	15.09							13.05	
**GDH**	11.16	9.39							19.98	17.99
**GOGAT**	0.14	0.33							0.16	
**OAT**	1.67								2.45	2.29
**P5CS**	0.68								0.80	0.61

Abreviation	Control	Control	Control	Control	Control	Control	Control	Control	NaCl	NaCl
**ProDH**	0.49								0.62	0.53

Parameter	NaCl	NaCl	NaCl	NaCl	NaCl	NaCl	NaCl	NaCl	No-W	No-W
**g_s**	0.02	0.03	0.01						0.00	0.00
**ET**		51.00			96.20	83.00			30.88	66.17
**Chlorophyll**	64.30		65.30		61.70	65.00	60.80	59.00	66.20	63.30
**Ψ_{leaf}**	-1.70	-1.70	-1.65						-1.60	-1.70
**Water content**	79.33	79.11	80.96	80.97	84.40	78.20	82.15	81.86	77.96	
**Water content**	77.90	77.40		79.79	81.72	75.65			62.31	
**Total biomass**		13.50	14.91	15.11	13.82				14.57	17.13
**Shoot biomass**	8.86	7.30	7.65	7.71	7.54				7.14	8.21
**Root biomass**		6.20	7.26	7.40	6.28				7.42	8.93
**Root/shoot ratio**		0.85	0.95	0.96	0.83				1.04	1.09
**Fructose**	9.92	2.23	5.54						17.31	9.52
**Glucose**	7.86	1.79	6.65						26.65	7.53
**Sucrose**	68.95	46.53	50.55						31.51	23.99
**Starch**	401.90	266.45	330.16						46.74	26.48
**Succinate**	0.10	0.15	0.12						0.10	0.09
**Malate**	2.54	9.12	6.57						3.92	4.66
**α-KG**	0.07	0.10	0.09						0.03	0.02
**Citrate**	2.64	3.72	5.78						0.71	0.60
**Sucrose**	21.91	6.39	8.96						21.07	17.60
**Fructose**	1.90	4.48	3.50						118.22	59.06
**Glucose**	2.87	3.42	4.20						94.65	47.34
**Sucrose**	22.81	28.75	33.82						38.70	48.56
**Starch**	4.83	15.67	4.38						4.05	3.67
**Succinate**	0.06	0.08	0.06						0.05	0.05
**Malate**	0.48	0.28	0.27						1.21	1.03
**α-KG**	0.01	0.01	0.01						0.03	0.04
**Citrate**	0.25	0.40	0.37						0.42	0.46
**Total**	122.18	133.99	141.36						173.94	155.79
**Total**	26.30								22.20	20.89
**Arg**	0.32	0.21	0.47						0.85	1.02
**Lys**	0.67	0.50	0.62						1.15	1.11
**Leu**	0.74	0.42	0.70						1.29	1.32
**Ile**	0.96	0.60	0.85						1.71	1.52
**Met**	0.13	0.13	0.19						0.28	0.37
**Phe**	1.49	0.72	1.01						2.00	2.49
**Trp**	1.19	0.48	0.93						1.46	1.66
**His**	1.13	0.78	1.13						1.47	1.62
**Tyr**	0.33	0.26	0.43						0.70	0.55
**Val**	2.64	1.60	2.56						5.35	4.66
**Gln**	0.53	0.20	0.82						1.52	1.26
**Pro**	26.02	24.17	23.04						15.04	17.36
**Asn**	18.81	11.48	36.83						51.49	48.76
**GABA**	0.57	0.30	0.39						0.50	0.87
**Thr**	1.83	1.86	2.87						3.82	3.54
**Ser**	20.18	12.29	19.85						2.71	4.99
**Gly**	0.28	0.36	0.28						0.40	0.36
**Ala**	1.50	4.26	1.48						2.05	2.40
**Glu**	7.94	11.98	15.42						11.35	10.66
**Asp**	2.27	4.37	4.90						3.97	3.64
**Total**	89.55	77.00	114.78						109.11	110.14
**Arg**	0.09	0.13	0.13						0.04	0.04
**Lys**	0.20	0.31	0.19						0.26	0.20

Abreviation	NaCl	NaCl	NaCl	NaCl	NaCl	NaCl	NaCl	NaCl	No-W	No-W
Leu	0.10	0.18	0.19						0.09	0.07
Ile	0.10	0.13	0.11						0.15	0.14
Met	0.03	0.02	0.03						0.01	0.01
Phe	0.08	0.09	0.11						0.08	0.08
Trp	0.01	0.02	0.01						0.01	0.01
His	0.03	0.06	0.08						0.09	0.07
Tyr	0.08	0.12	0.10						0.06	0.05
Val	0.28	0.41	0.31						0.56	0.53
Gln	1.22	0.47	0.64						0.18	0.15
Pro	3.51	5.31	2.49						1.14	1.24
Asn	6.86	5.93	7.83						3.10	2.90
GABA	1.44	2.34	1.18						0.75	0.57
Thr	0.30	0.41	0.36						0.21	0.19
Ser	0.75	1.36	1.86						0.37	0.46
Gly	0.07	0.07	0.06						0.06	0.05
Ala	0.64	0.48	0.24						0.27	0.20
Glu	0.64	0.52	0.74						0.58	0.50
Asp	1.02	1.95	0.59						1.78	1.38
Total	17.43	20.30	17.23						9.79	8.85
Arg	0.33	0.34	0.12						0.35	0.44
Lys	0.34	0.40	0.17						0.58	0.80
Leu	0.14	0.24	0.15						0.53	0.55
Ile	0.17	0.28	0.19						0.91	1.20
Met	0.04	0.07	0.04						0.10	0.12
Phe	0.15	0.24	0.14						0.41	0.55
Trp	0.38	0.44	0.20						0.70	1.03
His	1.10	1.43	0.34						2.09	2.01
Tyr	0.10	0.17	0.08						0.22	0.28
Val	0.49	0.71	0.45						2.23	2.50
Gln	0.63	0.87	0.57						0.51	0.66
Pro	7.09	11.44	5.32						11.85	15.61
Asn	23.81	20.40	5.05						42.32	37.84
GABA	0.74	1.20	0.74						1.19	1.30
Thr	0.91	0.95	0.79						1.87	2.20
Ser	1.75	1.92	1.48						3.46	3.62
Gly	0.22	0.34	0.22						0.57	0.43
Ala	0.51	0.71	0.57						1.10	1.08
Glu	0.99	1.25	0.94						1.24	1.25
Asp	0.43	0.54	0.44						1.00	0.80
Total	40.33	43.95	18.00						73.21	74.27
Na^+	0.51	0.43	0.48						0.02	0.02
NH_4^+	0.01	0.01	0.03						0.01	0.01
K^+	0.52	0.64	0.64						0.57	0.52
Mg^{2+}	0.21	0.26	0.21						0.20	0.19
Ca^{2+}	0.08	0.09	0.08						0.10	0.08
Cl^-	0.79	0.97	0.89	0.54	0.49	0.88	0.83	0.86	0.06	0.08
NO_3^-	0.05	0.08	0.08	0.05	0.01	0.02	0.08	0.07	0.03	0.02
SO_4^{2-}	0.27	0.16	0.21		0.24	0.27	0.15	0.18	0.39	0.39
PO_4^{3-}	0.03	0.05	0.05	0.02		0.04	0.02	0.04	0.05	0.04
Total	2.35	2.42	2.55						1.42	1.30
Na^+	0.81	0.91	0.65						0.05	0.06

Abreviation	NaCl	NaCl	NaCl	NaCl	NaCl	NaCl	NaCl	NaCl	No-W	No-W
NH_4^+	0.01	0.01	0.01						0.00	0.01
K^+	0.22	0.19	0.15						0.24	0.31
Mg^{2+}	0.01	0.01	0.01						0.04	0.07
Ca^{2+}	0.01	0.01	0.01						0.06	0.10
Cl^-	0.28		0.88	0.66	0.66		1.01	0.83	0.07	0.05
NO_3^-	0.01	0.01	0.00	0.00	0.01	0.00	0.01	0.00	0.02	0.01
SO_4^{2-}		0.09	0.09	0.08	0.06	0.03	0.08	0.09		0.48
PO_4^{3-}	0.00	0.01	0.01	0.01	0.01		0.01	0.01	0.02	0.02
Total	1.93	1.85	1.86						0.97	1.06
ASC	1.76								2.14	2.17
DHA	0.52								1.05	0.85
Total Asc	2.27								3.19	3.01
ASC/Total Asc	0.77								0.67	0.72
GSH	0.24								0.41	0.31
GSSG	0.29								0.47	0.36
Total Glut	0.53								0.88	0.66
GSH/Total Glut	0.46								0.47	0.46
hGSH	0.18								0.10	0.16
hGSSG	0.05								0.06	0.22
Total hGlut	0.22								0.15	0.38
hGSH/Total hGlu	0.79								0.64	0.42
ASC	0.13									
DHA	0.20									
Total Asc	0.33								0.18	
ASC/Total Asc	0.40									
GSH	0.14	0.06	0.04	0.05	0.05	0.07				
GSSG	0.08	0.08	0.11	0.08	0.16	0.11				
Total Glut	0.22	0.14	0.16	0.13	0.21	0.19				
GSH/Total Glut	0.63	0.43	0.27	0.37	0.24	0.39				
hGSH	0.18	0.11	0.12	0.09	0.11	0.10				
hGSSG	0.09	0.19	0.23	0.12	0.27	0.22				
Total hGlut	0.28	0.30	0.35	0.20	0.37	0.33				
hGSH/Total hGlu	0.67	0.37	0.33	0.42	0.29	0.31				
G6PDH	0.11	0.17							0.18	0.13
IDH	2.54	3.00	1.96						1.84	2.29
AAT	3.46	2.37	1.85						3.93	3.41
GDH	0.59	0.67	0.75						0.76	0.66
GOGAT	0.05								0.05	0.04
GS	0.03								0.03	0.03
OAT	0.75								0.39	0.40
P5CS	0.65								0.44	0.51
ProDH	0.13								0.19	0.22
AlkINV	4.11	1.69	1.67	0.59	3.87				3.78	3.50
G6PDH	6.51	6.53	7.46						2.49	2.69
IDH	7.48	10.26	7.28		7.44				5.88	6.09
SuSy	0.64	0.72	1.00						1.57	1.66
AAT	9.77	9.23	6.57						11.47	5.75
GDH	19.85	9.45							16.46	9.92
GOGAT	0.34	0.37							0.17	
OAT	3.09								2.08	2.37
P5CS	0.92								0.86	1.13

Abreviation	NaCl	NaCl	NaCl	NaCl	NaCl	NaCl	NaCl	NaCl	No-W	No-W
**ProDH**	0.61								0.89	0.76

Parameter	No-W	No-W	No-W	No-W	No-W	No-W	No-W	No-W	PEG	PEG
g_s	0.00	0.00	0.00						0.00	0.00
ET	43.77	69.16		41.46	36.88	29.67	45.00	91.90	36.78	
Chlorophyll	64.40	62.40	60.78						59.00	59.70
Ψ_{leaf}	-1.70	-1.70	-1.70						-1.60	-1.70
Water content		80.86	75.88	75.59	79.69	78.97	77.25	76.75		
Water content			60.01	62.37	67.48	71.68	67.46			
Total biomass	21.84	17.75	13.61						18.32	13.94
Shoot biomass	10.40	8.42	6.78						10.41	6.26
Root biomass	11.44	9.33	6.82						7.90	7.68
Root/shoot ratio	1.10	1.11	1.01						0.76	1.23
Fructose	24.90	7.95	17.65						4.98	14.33
Glucose	35.54	9.74	26.36						6.15	9.06
Sucrose	11.92	24.44	36.24						137.61	94.47
Starch	57.66	49.29	121.82						0.00	0.00
Succinate	0.13	0.13	0.08						0.09	0.06
Malate	6.09	5.01	4.11						2.68	3.48
α-KG	0.05	0.04	0.02						0.03	0.03
Citrate	2.03	1.00	0.86						0.44	0.68
Sucrose	13.08	18.31	20.21						15.43	16.61
Fructose	51.67	88.30	55.00						7.11	7.97
Glucose	42.23	75.35	53.48						7.55	9.45
Sucrose	44.80	39.19	45.00						46.01	40.49
Starch	3.28	3.55	1.72						4.67	4.70
Succinate	0.05	0.05	0.09						0.30	0.52
Malate	1.16	1.02	3.14						0.60	1.08
α-KG	0.04	0.03	0.04						0.02	0.03
Citrate	0.41	0.49	0.55						0.26	0.31
Total	101.87	122.27	148.32						202.76	107.71
Total	25.00								22.02	18.10
Arg	0.50	1.23	0.75						1.66	1.51
Lys	0.98	1.72	1.26						1.15	1.04
Leu	1.38	1.53	1.55						1.07	1.05
Ile	1.62	1.89	1.74						1.39	1.24
Met	0.28	0.27	0.34						0.29	0.29
Phe	1.74	2.17	2.00						3.78	3.60
Trp	1.03	1.53	1.22						2.26	2.04
His	0.80	1.64	1.10						2.97	2.87
Tyr	0.63	0.89	0.83						0.80	0.72
Val	4.52	5.38	5.12						5.69	6.01
Gln	1.13	1.46	1.69						1.98	1.43
Pro	15.82	13.77	18.34						65.20	68.93
Asn	45.67	83.34	56.87						44.21	49.42
GABA	0.71	0.70	0.87						0.82	0.90
Thr	3.29	3.89	4.38						1.38	1.39
Ser	2.55	5.20	2.38						5.43	6.82
Gly	0.41	0.38	0.54						0.49	0.64
Ala	2.79	2.51	3.19						2.41	2.15
Glu	13.98	15.99	13.04						8.00	7.73
Asp	3.92	3.74	3.40						2.06	1.63
Total	103.74	149.22	120.61						153.06	161.43
Arg	0.04	0.04	0.08						0.05	0.08
Lys	0.17	0.23	0.29						0.14	0.19

Abreviation	No-W	No-W	No-W	No-W	No-W	No-W	No-W	No-W	PEG	PEG
**Leu**	0.07	0.08	0.08						0.06	0.10
**Ile**	0.13	0.13	0.15						0.08	0.10
**Met**	0.01	0.01	0.02						0.01	0.02
**Phe**	0.06	0.06	0.09						0.06	0.09
**Trp**	0.01	0.01	0.02						0.01	0.01
**His**	0.04	0.04	0.06						0.11	0.15
**Tyr**	0.05	0.06	0.06						0.07	0.11
**Val**	0.49	0.53	0.54						0.40	0.54
**Gln**	0.19	0.18	0.19						0.40	0.45
**Pro**	1.28	0.97	1.74						0.98	2.29
**Asn**	2.94	4.62	4.04						8.31	9.46
**GABA**	0.85	0.79	0.67						1.27	1.61
**Thr**	0.19	0.24	0.23						0.22	0.33
**Ser**	0.16	0.45	0.28						0.35	0.73
**Gly**	0.04	0.04	0.04						0.05	0.08
**Ala**	0.37	0.25	0.35						0.21	0.41
**Glu**	0.66	0.61	1.16						0.39	0.77
**Asp**	1.89	1.89	2.64						1.27	3.00
**Total**	9.63	11.24	12.74						14.45	20.50
**Arg**	0.31	0.55	0.41						0.34	0.37
**Lys**	0.56	0.82	0.72						0.39	0.41
**Leu**	0.52	0.38	0.61						0.44	0.50
**Ile**	0.91	0.68	1.10						0.90	0.93
**Met**	0.10	0.10	0.12						0.09	0.10
**Phe**	0.42	0.51	0.67						0.20	0.20
**Trp**	0.70	1.02	0.99						0.48	0.51
**His**	1.58	2.71	1.57						3.11	3.06
**Tyr**	0.21	0.38	0.26						0.19	0.21
**Val**	2.11	1.75	2.40						1.69	1.83
**Gln**	0.39	1.79	0.56						0.29	0.27
**Pro**	10.92	14.36	15.08						13.15	11.45
**Asn**	43.40	42.68	54.07						17.42	20.11
**GABA**	1.18	1.00	1.25						2.64	2.39
**Thr**	2.82	1.98	2.71						1.63	1.91
**Ser**	3.87	3.26	3.48						2.57	2.87
**Gly**	0.39	0.62	0.49						0.85	0.55
**Ala**	1.43	1.33	1.00						4.03	2.16
**Glu**	1.01	1.72	1.64						0.54	0.82
**Asp**	0.72	1.29	1.20						0.45	0.53
**Total**	73.53	78.91	90.34						51.38	51.19
**Na^+**	0.02	0.02	0.02						0.02	0.02
**NH_4^+**	0.01	0.02	0.01						0.01	0.01
**K^+**	0.49	0.56	0.58						0.35	0.40
**Mg^{2+}**	0.19	0.20	0.17						0.12	0.14
**Ca^{2+}**	0.09	0.07	0.06						0.04	0.04
**Cl^-**	0.08		0.10	0.08	0.08	0.11	0.10	0.08	0.10	0.07
**NO_3^-**	0.07	0.07	0.07	0.02	0.03	0.06	0.07	0.05	0.02	0.04
**SO_4^{2-}**	0.27		0.29	0.40	0.42	0.26	0.36	0.31	0.26	0.21
**PO_4^{3-}**	0.05	0.08	0.08	0.03		0.04	0.08	0.04	0.04	0.04
**Total**	1.33	1.40	1.39						0.92	0.98
**Na^+**	0.02	0.09	0.09						0.02	0.01

Abreviation	No-W	No-W	No-W	No-W	No-W	No-W	No-W	No-W	PEG	PEG
NH_4^+	0.00	0.02	0.01						0.00	0.01
K^+	0.10	0.40	0.40						0.11	0.13
Mg^{2+}	0.04	0.04	0.06						0.02	0.01
Ca^{2+}	0.02	0.09	0.11						0.00	0.02
Cl^-	0.07	0.08	0.06	0.09	0.10	0.08	0.05	0.06	0.02	0.02
NO_3^-	0.04	0.02	0.02	0.01	0.02	0.03	0.01	0.04	0.01	0.01
SO_4^{2-}	0.39	0.39	0.41	0.42	0.50	0.43	0.39	0.34	0.04	0.05
PO_4^{3-}	0.02	0.02	0.02	0.02	0.02	0.02	0.02	0.02	0.03	0.02
Total	0.71	1.24	1.13						0.25	0.27
ASC	1.89								1.43	1.51
DHA	0.92								0.74	1.00
Total Asc	2.81								2.17	2.51
ASC/Total Asc	0.67								0.66	0.60
GSH	0.23								0.25	0.17
GSSG	0.23								0.58	0.45
Total Glut	0.46								0.83	0.61
GSH/Total Glut	0.50								0.30	0.27
hGSH	0.13								0.12	0.09
hGSSG	0.14								0.07	0.10
Total hGlut	0.27								0.19	0.18
hGSH/Total hGlu	0.48								0.61	0.48
ASC										
DHA										
Total Asc		0.34		0.22	0.20	0.23	0.20	0.22	0.19	
ASC/Total Asc										
GSH				0.15	0.18	0.31	0.25	0.06	0.04	0.02
GSSG				0.17	0.31		0.24	0.45	0.04	0.09
Total Glut				0.32	0.49		0.49	0.51	0.08	0.11
GSH/Total Glut				0.46	0.37		0.51	0.11	0.50	0.17
hGSH				0.21	0.39	0.27	0.53	0.47	0.08	0.04
hGSSG				0.68	1.01	0.31	1.41	0.88	0.14	0.22
Total hGlut				0.89	1.40	0.58	1.94	1.35	0.21	0.27
hGSH/Total hGlut				0.23	0.28	0.47	0.27	0.35	0.36	0.16
G6PDH	0.16	0.25							0.08	0.06
IDH	1.67	2.66	1.85						2.37	1.91
AAT	3.48	2.71	2.74						4.93	3.82
GDH	0.85	0.86	0.90						1.00	0.68
GOGAT	0.03								0.07	0.04
GS	0.03								0.02	0.03
OAT	0.47								0.14	0.14
P5CS	0.41								0.43	0.41
ProDH	0.19								0.22	0.27
AlkINV	4.14	4.43	3.60	3.18	3.50				0.88	0.74
G6PDH	2.79	2.66							0.85	3.48
IDH	7.14	7.73	5.44		5.31				6.20	6.50
SuSy		0.31	0.38						1.93	2.20
AAT	5.42	4.95	10.91						12.01	8.86
GDH	11.16	9.26							16.17	9.80
GOGAT	0.15	0.28							0.11	
OAT	2.24								2.14	2.82
P5CS	1.12								1.12	1.26

Abreviation	No-W	No-W	No-W	No-W	No-W	No-W	No-W	No-W	PEG	PEG
**ProDH**	1.08								0.81	0.99

Parameter	PEG	PEG	PEG	PEG	PEG	PEG	PEG	PEG
g_s	0.00	0.00	0.00					
ET	58.00					44.00	65.00	47.50
Chlorophyll	57.50	59.30	58.50					
Ψ_{leaf}	-1.70	-1.70	-1.70					
Water content	63.87	68.36	70.34	70.73	69.76			
Water content	79.31	88.87	85.06	82.85	81.64			
Total biomass	13.80	14.45	13.62					
Shoot biomass	7.40	7.56	7.42					
Root biomass	6.40	6.89	6.20					
Root/shoot ratio	0.86	0.91	0.83					
Fructose	14.03	5.79	5.33					
Glucose	17.06	5.05	7.99					
Sucrose	83.01	58.49	40.33					
Starch	0.00	0.00	0.00					
Succinate	0.07	0.08	0.09					
Malate	2.52	3.10	2.72					
α-KG	0.03	0.04	0.03					
Citrate	0.42	0.49	0.42					
Sucrose	14.00	23.49	17.35					
Fructose	8.98	1.73	9.21					
Glucose	13.42	2.13	8.38					
Sucrose	52.14	31.78	56.17					
Starch	5.07		9.39					
Succinate	0.69	0.55	0.69					
Malate	0.97	0.84	0.69					
α-KG	0.03	0.03	0.04					
Citrate	0.37	0.32	0.33					
Total	143.03	168.84	138.32					
Total	23.50							
Arg	1.28	0.47	0.90					
Lys	0.76	0.80	0.75					
Leu	0.65	0.64	0.69					
Ile	0.88	0.72	0.96					
Met	0.22	0.11	0.17					
Phe	2.52	2.65	2.13					
Trp	1.59	1.70	1.10					
His	2.93	1.54	1.61					
Tyr	0.48	0.47	0.52					
Val	5.10	4.30	4.60					
Gln	1.60	0.26	0.92					
Pro	40.44	61.44	47.48					
Asn	42.52	26.21	49.60					
GABA	0.46	0.88	0.64					
Thr	1.32	1.14	1.55					
Ser	6.58	5.00	4.49					
Gly	0.40	0.55	0.43					
Ala	1.78	2.00	1.64					
Glu	6.37	8.96	6.04					
Asp	2.25	1.96	1.89					
Total	120.14	121.79	128.13					
Arg	0.04	0.15	0.06					
Lys	0.12	0.32	0.17					

Abreviation	PEG	PEG	PEG	PEG	PEG	PEG	PEG	PEG
**Leu**	0.06	0.17	0.08					
**Ile**	0.06	0.16	0.10					
**Met**	0.01	0.03	0.01					
**Phe**	0.05	0.16	0.08					
**Trp**	0.01	0.02	0.02					
**His**	0.02	0.09	0.04					
**Tyr**	0.06	0.16	0.09					
**Val**	0.37	0.65	0.51					
**Gln**	0.29	0.35	0.39					
**Pro**	1.71	3.90	2.12					
**Asn**	3.62	10.81	7.26					
**GABA**	0.92	2.21	1.09					
**Thr**	0.18	0.49	0.28					
**Ser**	0.43	0.55	0.64					
**Gly**	0.06	0.09	0.05					
**Ala**	0.32	0.63	0.31					
**Glu**	0.57	1.34	0.56					
**Asp**	1.57	4.70	2.01					
**Total**	10.47	26.96	15.89					
**Arg**	0.64	0.15	0.65					
**Lys**	0.90	0.16	0.88					
**Leu**	0.73	0.21	0.77					
**Ile**	1.13	0.47	1.52					
**Met**	0.14	0.06	0.13					
**Phe**	0.64	0.41	0.36					
**Trp**	0.76	0.25	0.98					
**His**	4.18	0.91	4.29					
**Tyr**	0.45	0.12	0.42					
**Val**	2.45	0.94	2.90					
**Gln**	0.98	0.17	0.74					
**Pro**	16.65	12.38	17.63					
**Asn**	42.05	14.26	30.58					
**GABA**	5.14	2.14	4.04					
**Thr**	2.72	1.15	3.12					
**Ser**	4.09	2.29	3.96					
**Gly**	0.90	0.42	1.11					
**Ala**	4.26	3.16	3.82					
**Glu**	1.45	1.35	1.34					
**Asp**	0.84	0.69	0.68					
**Total**	91.11	41.69	79.92					
**Na^+**	0.01	0.01	0.01					
**NH_4^+**	0.01	0.01	0.02					
**K^+**	0.44	0.37	0.45					
**Mg^{2+}**	0.14	0.13	0.13					
**Ca^{2+}**	0.08	0.05	0.06					
**Cl^-**	0.05	0.10	0.08	0.08	0.07	0.06	0.10	0.07
**NO_3^-**	0.03		0.02	0.02	0.03	0.03	0.04	0.02
**SO_4^{2-}**	0.32	0.16	0.36	0.22	0.23	0.32		0.30
**PO_4^{3-}**	0.04	0.04	0.05	0.02	0.02	0.02	0.02	0.03
**Total**	1.10	0.98	1.11					
**Na^+**	0.02	0.01	0.01					

Abreviation	PEG	PEG	PEG	PEG	PEG	PEG	PEG	PEG
NH_4^+	0.01	0.01	0.01					
K^+	0.16	0.09	0.11					
Mg^{2+}	0.02	0.01	0.01					
Ca^{2+}	0.02	0.02	0.03					
Cl^-	0.02	0.02	0.03	0.02	0.04	0.03	0.02	0.03
NO_3^-	0.00	0.01	0.00	0.00	0.01	0.00	0.01	0.01
SO_4^{2-}	0.05	0.03	0.07	0.04	0.05	0.06	0.03	0.08
PO_4^{3-}	0.02	0.01	0.03	0.02	0.02	0.02	0.01	0.02
Total	0.34	0.24	0.28					
ASC	1.56							
DHA	0.91							
Total Asc	2.47							
ASC/Total Asc	0.63							
GSH	0.28							
GSSG	0.54							
Total Glut	0.82							
GSH/Total Glut	0.34							
hGSH	0.14							
hGSSG	0.04							
Total hGlut	0.18							
hGSH/Total hGlut	0.77							
ASC								
DHA								
Total Asc			0.27				0.29	
ASC/Total Asc								
GSH	0.05	0.07			0.04	0.17	0.05	
GSSG	0.14	0.15				0.41	0.02	
Total Glut	0.20	0.23				0.57	0.07	
GSH/Total Glut	0.26	0.32				0.29	0.75	
hGSH	0.16	0.19			0.10	0.19	0.07	
hGSSG	0.28	0.08				0.35	0.13	
Total hGlut	0.43	0.27				0.54	0.20	
hGSH/Total hGlut	0.36	0.70				0.35	0.35	
G6PDH	0.09	0.20						
IDH	1.59	1.83	1.59					
AAT	3.41	3.41	3.39					
GDH	0.63	0.98	0.95					
GOGAT	0.05							
GS	0.03							
OAT	0.17							
P5CS	0.49							
ProDH	0.22							
AlkINV	1.47	0.79	1.52	1.71	3.97			
G6PDH	3.35	2.83						
IDH	6.74	7.26	12.88		4.37			
SuSy	0.31	0.49	0.00					
AAT	9.95	6.07						
GDH	12.98							
GOGAT	0.18	0.23						
OAT	2.59							
P5CS	1.10							

Abreviation	PEG	PEG	PEG	PEG	PEG	PEG	PEG	PEG
**ProDH**	0.94							
