# Supplementary figures and images for: Strategies to Apply Water-Deficit Stress: Similarities and Disparities at the Whole Plant Metabolism Level in Medicago truncatula
Source: Int J Mol Sci. 2021 Mar 10;22(6):2813. doi: 10.3390/ijms22062813 (PMC8002188; doi:10.3390/ijms22062813)

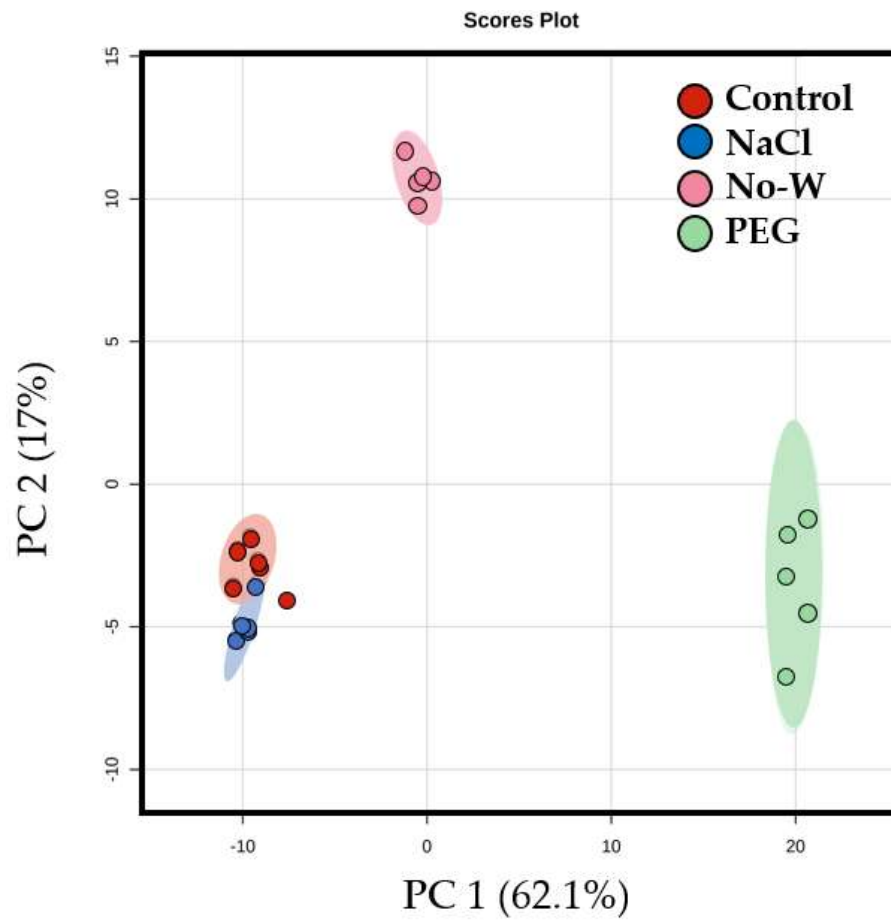

Supplement: Supplementary file 1 [file ijms-22-02813-s001.zip › ijms-1103292-revision-suppl/ijms-revision-figure S2.pdf]
